# Citizen science project reveals high diversity in Didymellaceae (Pleosporales, Dothideomycetes)

**DOI:** 10.3897/mycokeys.65.47704

**Published:** 2020-03-10

**Authors:** Lingwei Hou, Margarita Hernández-Restrepo, Johannes Zacharias Groenewald, Lei Cai, Pedro W. Crous

**Affiliations:** 1 State Key Laboratory of Mycology, Institute of Microbiology, Chinese Academy of Sciences, Beijing 100101, China Institute of Microbiology, Chinese Academy of Sciences Beijing China; 2 University of Chinese Academy of Sciences, Beijing 100049, China University of Chinese Academy of Sciences Beijing China; 3 Westerdijk Fungal Biodiversity Institute, Uppsalalaan 8, 3584 CT Utrecht, The Netherlands Westerdijk Fungal Biodiversity Institute Utrecht Netherlands; 4 Microbiology, Department of Biology, Utrecht University, Padualaan 8, 3584 CH Utrecht, The Netherlands Utrecht University Utrecht Netherlands

**Keywords:** biodiversity, new taxa, *
Phoma
*, phylogeny, soil-borne fungi

## Abstract

Fungal communities play a crucial role in maintaining the health of managed and natural soil environments, which directly or indirectly affect the properties of plants and other soil inhabitants. As part of a Citizen Science Project initiated by the Westerdijk Fungal Biodiversity Institute and the Utrecht University Museum, which aimed to describe novel fungal species from Dutch garden soil, the diversity of Didymellaceae, which is one of the largest families in the Dothideomycetes was investigated. A preliminary analysis of the ITS and LSU sequences from the obtained isolates allowed the identification of 148 strains belonging to the family. Based on a multi-locus phylogeny of a combined ITS, LSU, *rpb2* and *tub2* alignment, and morphological characteristics, 20 different species were identified in nine genera, namely *Ascochyta*, *Calophoma*, *Didymella*, *Juxtiphoma*, *Nothophoma*, *Paraboeremia*, *Phomatodes*, *Stagonosporopsis*, and *Xenodidymella*. Several isolates confirmed to be ubiquitous plant pathogens or endophytes were for the first time identified from soil, such as *Ascochyta syringae*, *Calophoma clematidis*-*rectae*, and *Paraboeremia litseae*. Furthermore, one new genus and 12 novel species were described from soil: *Ascochyta benningiorum***sp. nov.**, *Didymella degraaffiae***sp. nov.**, *D. kooimaniorum***sp. nov.**, *Juxtiphoma kolkmaniorum***sp. nov.**, *Nothophoma brennandiae***sp. nov.**, *Paraboeremia rekkeri***sp. nov.**, *P. truiniorum***sp. nov.**, *Stagonosporopsis stuijvenbergii***sp. nov.**, *S. weymaniae***sp. nov.**, *Vandijckomycella joseae***gen. nov. et sp. nov.**, *V. snoekiae***sp. nov.**, and *Xenodidymella weymaniae***sp. nov**. From the results of this study, soil was revealed to be a rich substrate for members of Didymellaceae, several of which were previously known only from diseased or apparently healthy plant hosts.

## Introduction

Due to high plasticity and the capacity to adapt and survive in adverse or unfavourable conditions, fungi are exceedingly successful soil inhabitants (Frąc et al. 2018). The majority of the fungal species presently known can survive in, or directly adapt to, the soil environment (Bridge and Spooner 2001; Botha 2011). Soil-borne fungi play essential roles in nutrient cycling in terrestrial ecosystems and are able to break down all kinds of organic matter, decompose soil components or act as effective biosorbents of toxic metals, thereby helping to maintain soil health (Anderson and Domsch 1973; Bender et al. 2013; Rudgers et al. 2014; Tedersoo et al. 2014; Yang et al. 2017; Frąc et al. 2018). Soil fungal communities also form symbiotic associations with plants, thereby improving nutrient absorption (Voøíšková and Baldrian 2012). Most fungal taxa found in the soil are continuously present in the environment as harmless saprobic organisms, but some also play a negative role. For instance, plant pathogenic fungi in soil could infect seedlings or other plant tissues when conditions are suitable, resulting in significant damage (van Agtmaal et al. 2017). In addition, some fungi reside in soil in the form of propagules to survive in an unsuitable environment, posing a long-term threat to other inhabitants (Maryani et al. 2019).

Didymellaceae is a ubiquitous fungal family including saprobic, endophytic and pathogenic species (Aveskamp et al. 2008, 2010; Marin-Felix et al. 2017). More than 50% of the species in this family have been reported as plant pathogens, causing great losses to a wide range of economic crops (Aveskamp et al. 2008). Other species are found in different substrates, including soil, air, and water or cyst nematodes (Dorenbosch 1970; Chen et al. 1996; Boerema et al. 2004; Aveskamp et al. 2010; Porras-Alfaro et al. 2011; Chen et al. 2015, 2017; Grishkan 2018; Valenzuela-Lopez et al. 2018), and even in some extreme environments such as deep-sea sediments, or soils in Antarctica, deserts, and karst caves (Ruisi et al. 2007; Li et al. 2016; Zhang et al. 2016a, 2016b, 2017; Chen et al. 2017; Nagano et al. 2017; Grishkan 2018). Although recent research has suggested that the soil environment represents an important niche for the discovery of novel phoma-like species (Chen et al. 2017, van Agtmaal et al. 2017), very few studies have investigated the diversity of Didymellaceae in soil, which is a massive reservoir for plant, animal and human pathogens.

The first paper systematically investigating Didymellaceae species from soil was published by Dorenbosch (1970), who provided diagnostic characteristics and a usable identification method (keys) for nine ubiquitous phoma-like fungi from soil, including *Pyrenochaeta acicola*, *Phoma chrysanthemicola*, *Ph. eupyrena*, *Ph. exigua*, *Ph. fimeti*, *Ph. glomerata*, *Ph. herbarum*, Ph. medicaginis var. pinodella, and *Ph. prunicola* (names used at that time). Later, Boerema et al. (2004) and Domsch et al. (2007) illustrated several Didymellaceae species from soil and provided their ecological distributions. Since then, a few species have been reported sporadically, along with the research of root and seed diseases, but studies of Didymellaceae from soil are still rare, with even fewer describing new taxa from soil. Most species in previous studies have been reallocated to other genera in this family based on their DNA phylogeny (Chen et al. 2015, 2017; Valenzuela-Lopez et al. 2018). To date, only approximately 30 species from eight genera in Didymellaceae have been recorded from soil, namely *Ascochyta*, *Phoma*, *Didymella*, *Neodidymelliopsis*, *Epicoccum*, *Cumuliphoma*, *Ectophoma* and *Juxtiphoma* (Dorenbosch 1970; Boerema et al. 2004; Domsch et al. 2007; Chen et al. 2017; Valenzuela-Lopez et al. 2018). Although most of the species documented from soil are plant-associated (pathogens and endophytes), some species, such as *Ph. herbarum* and *J. eupyrena*, are characterised as soil-borne (Dorenbosch 1970; Boerema et al. 2004).

Didymellaceae species from soil always produce diverse metabolites, some of which can be cytotoxic, including cytochalasin A and B, deoxaphomin, proxiphomin and tenuazonic acid (Bennett et al. 2018). Currently, most Didymellaceae species thus far found in the soil environment were originally described from plant substrates, such as leaves, seedlings, wood, stem bases or roots, some of which are even capable of wood decay (Boerema et al. 2004; Aveskamp et al. 2008, 2010; Chen et al. 2015). On the contrary, crops that are grown in close proximity to infected soil appear to be more contaminated, given that soil is a known source of plant pathogenic fungi (Paterson and Lima 2017). Besides, some species have also been reported to be opportunistic pathogens in animals and humans, such as *J. eupyrena* (= *Phoma eupyrena*) and *Phoma herbarum* (Bakerspigel et al. 1981; Tullio et al. 2010). Considering the potential threat and great losses caused by soil-borne pathogens, and the application in the biotechnological or pharmaceutical industries, knowledge of the diversity of Didymellaceae in soil is urgently needed to better understand the functions, interactions and ecosystem feedback of fungi in the terrestrial environment.

The present Citizen Science Project was initiated by the Westerdijk Fungal Biodiversity Institute (WI) and the Utrecht University Museum, aiming to investigate the diversity of fungi in Dutch garden soil collected by children in their home gardens from different regions in the Netherlands (Groenewald et al. 2018). During the course of this project thousands of isolates were obtained from 293 soil samples. Of these, 148 isolates were found to belong to Didymellaceae, and subsequently selected for study. The aim of the present study was to investigate the diversity of Didymellaceae from Dutch garden soil, describe and illustrate novel species, and compare them with known and related species.

## Materials and methods

### Sampling and isolation

Protocols for the collection and processing of soil samples are described in Groenewald et al. (2018) and Giraldo et al. (2019). Isolates are maintained in the Johanna Westerdijk (**JW**) working collection housed at the **WI** in Utrecht, the Netherlands. New and interesting strains were also deposited in the **CBS** fungal collection and holotypes in the fungarium at the **WI**, respectively.

### DNA extraction, PCR amplification and sequencing

Genomic DNA was extracted using the Wizard® Genomic DNA Purification Kit (Promega, Madison, USA) following the manufacturer’s protocols. Initially, the internal transcribed spacer regions 1 and 2 and 5.8S nuclear ribosomal RNA gene (ITS) and partial large subunit nrDNA (LSU) were amplified using primer pairs ITS5/ITS4 (White et al. 1990) and LR0R/LR5 (Vilgalys and Hester 1990; Vilgalys and Sun 1994), respectively. For members of Didymellaceae two extra loci were amplified, the partial beta-tubulin (*tub2*) and the partial RNA polymerase II second largest subunit (*rpb2*), using the primer pairs Tub2Fd/Tub4Rd (Woudenberg et al. 2009) and Rpb2-5F2/Rpb2-7cR (Liu et al. 1999; Sung et al. 2007), respectively. The PCR amplifications were performed following Chen et al. (2015), except for *rpb2*, which was amplified in a total volume of 12.5 µL containing 1.25 µL of 10× EasyTaq Buffer (Bioline, Luckenwalde, Germany), 0.5 µL of dNTPs (40 μM), 0.5 µL of MgCl_2_ (2 mM), 0.5 µL of bovine berum albumin (BSA, 1 μg/μL), 0.5 µL of each primer (0.2 μM), 0.1 µL of Taq DNA polymerase (Bioline) and 1 µL of genomic DNA. PCR conditions for *rpb2* were set as follows: an initial denaturation at 94 °C for 5 min; 35 cycles of denaturation at 95 °C for 45 s, annealing at 55 °C for 80 s and extension at 72 °C for 2 min; and a final extension step at 72 °C for 10 min. The PCR products were sequenced according to the methods of Crous et al. (2013). Consensus sequences were assembled from forward and reverse sequences using Seqman Pro v.12.1.0 (DNASTAR, Madison, WI, USA). All sequences generated in this study were deposited in GenBank (Table 1).

**Table 1.** Taxa used in this study and their GenBank accession numbers.

Taxon name^1^	Strain number^2^	Substrate	Country	GenBank Accession numbers^3^			
				*rpb2*	*tub2*	ITS	LSU
*Allophoma cylindrispora*	CBS 142453^T^; FMR 13723	Human superficial tissue	USA	LT593058	LT592989	LN907376	LT592920
*Al. nicaraguensis*	CBS 506.91^T^; IMI 215229; PD 91/876	*Coffea* sp.	Nicaragua	KT389551	GU237596	GU238058	GU237876
*Al. piperis*	CBS 268.93^T^; PD 88/720	*Peperomia pereskifolia*	The Netherlands	KT389554	GU237644	GU238129	GU237816
*Al. tropica*	CBS 436.75^T^	*Saintpaulia ionantha*	Germany	KT389556	GU237663	GU238149	GU237864
***Ascochyta benningiorum***	**CBS 144957^T^; JW 196005**	Garden soil	The Netherlands	**MN824606**	**MN824755**	**MN823432**	**MN823581**
	**CBS 144958; JW 196023**	Garden soil	The Netherlands	**MN824607**	**MN824756**	**MN823433**	**MN823582**
	**JW 196013**	Garden soil	The Netherlands	**MN824608**	**MN824757**	**MN823434**	**MN823583**
*A. boeremae*	CBS 372.84^T^; PD 80/1246	*Pisum sativum*	Australia	–	KT389774	KT389697	KT389480
	CBS 373.84; PD 80/1247	*Pisum sativum*	Australia	KT389560	KT389775	KT389698	KT389481
*A. fabae*	CBS 649.71	*Vicia faba*	The Netherlands	–	GU237527	GU237964	GU237902
	CBS 524.77	*Phaseolus vulgaris*	Belgium	–	GU237526	GU237963	GU237880
	PD 83/492	*Phaseolus vulgaris*	The Netherlands	–	GU237528	GU237965	GU237917
*A. herbicola*	CBS 629.97^R^; PD 76/1017	Water	USA	KP330421	GU237614	GU238083	GU237898
*A. lentis*	CBS 370.84; PD 81/783	*Lens culinaris*	Unknown	–	KT389768	KT389691	KT389474
A. medicaginicola var. macrospora	CBS 112.53^T^	*Medicago sativa*	USA	–	GU237628	GU238101	GU237749
	CBS 404.65^R^; IMI 116999	*Medicago sativa*	Canada	KP330423	GU237629	GU238102	GU237859
*A. nigripycnidia*	CBS 116.96^T^; PD 95/7930	*Vicia cracca*	Russia	–	GU237637	GU238118	GU237756
*A. phacae*	CBS 184.55^T^	*Phaca alpina*	Switzerland	–	KT389769	KT389692	KT389475
*A. pisi*	CBS 126.54	*Pisum sativum*	The Netherlands	DQ677967	GU237531	EU754137	GU237772
	CBS 122785^T^; PD 78/517	*Pisum sativum*	The Netherlands	–	GU237532	GU237969	GU237763
	CBS 122751; ATCC 201620	*Pisum sativum*	Canada	EU874867	KP330388	KP330444	KP330432
*A. rabiei*	CBS 534.65	*Cicer arietinum*	India	KP330405	GU237533	GU237970	GU237886
	CBS 237.37^T^	*Cicer arietinum*	Bulgaria	–	KT389773	KT389696	KT389479
*A. syringae*	CBS 545.72	*Syringa vulgaris*	The Netherlands	–	KT389777	KT389700	KT389483
	JW 1074	Garden soil	The Netherlands	**MN824605**	**MN824754**	**MN823431**	**MN823580**
*A. versabilis*	CBS 876.97^R^	*Silene* sp.	The Netherlands	KT389561	GU237664	GU238152	GU237909
*A. viciae*	CBS 451.68	*Vicia sepium*	The Netherlands	KT389562	KT389778	KT389701	KT389484
*A. viciae-pannonicae*	CBS 254.92	*Vicia pannonica*	Czech Republic	–	KT389779	KT389702	KT389485
Boeremia exigua var. heteromorpha	CBS 443.94^T^	*Nerium oleander*	Italy	KT389573	GU237497	GU237935	GU237866
B. exigua var. populi	CBS 100167^T^; PD 93/217	*Populus* (×)*euramericana*	The Netherlands	–	GU237501	GU237939	GU237707
*Briansuttonomyces eucalypti*	CBS 114879^T^	*Eucalyptus* sp.	South Africa	–	KU728595	KU728519	KU728479
	CBS 114887	*Eucalyptus* sp.	South Africa	–	KU728596	KU728520	KU728480
*Calophoma clematidina*	CBS 102.66	*Clematis* sp.	UK	KT389587	FJ427099	FJ515630	FJ426988
	CBS 108.79^T^; PD 78/522	*Clematis* sp.	The Netherlands	KT389588	FJ427100	FJ515632	FJ426989
*C. clematidis-rectae*	JW 179007	Garden soil	The Netherlands	**MN824612**	**MN824761**	**MN823438**	**MN823587**
	CBS 507.63	*Clematis* sp.	The Netherlands	KT389589	FJ515624	FJ515647	FJ515606
*C. vodakii*	CBS 173.53^T^	*Hepatica triloba*	Switzerland	–	KT389791	KT389714	KT389497
*Coniothyrium palmarum*	CBS 400.71	*Chamaerops humilis*	Italy	KT389592	KT389792	EU754153	AY720708
*Cumuliphoma indica*	CBS 654.77^T^; FMR 15341	Unknown	India	LT623261	FJ427153	GU238122	FJ427043
*Cu. omnivirens*	CBS 341.86^T^; FMR 14915	*Phaseolus vulgaris*	Belgium	LT62326	FJ427152	LT623214	FJ427042
*Cu. pneumoniae*	CBS 142454^T^; FMR13739	Human respiratory tract	USA	LT593063	LT592994	LN907392	LT592925
*Didymella aeria*	CGMCC 3.18353^T^; LC 7441	Air	China	KY742137	KY742293	KY742205	KY742051
*D. aliena*	LC 8121	*Pyrus calleryana*	Italy	–	KY742295	KY742207	KY742053
	CBS 379.93; PD 82/945	*Berberis* sp.	The Netherlands	KP330416	GU237578	GU238037	GU237851
*D. americana*	CBS 185.85^R^; PD 80/1191	*Zea mays*	USA	KT389594	FJ427088	GU237990	FJ426972
*D. anserina*	CBS 360.84^R^	Potato flour	The Netherlands	KT389596	GU237551	GU237993	GU237839
*D. aquatica*	CGMCC 3.18349^T^; LC 5556	Water	China	KY742140	KY742297	KY742209	KY742055
*D. arachidicola*	CBS 333.75^T^; ATCC 28333; IMI 386092	*Arachis hypogaea*	South Africa	KT389598	GU237554	GU237996	GU237833
*D. aurea*	CBS 269.93^T^; PD 78/1087	*Medicago polymorpha*	New Zealand	KT389599	GU237557	GU237999	GU237818
*D. bellidis*	CBS 714.85^R^; PD 74/265	*Bellis perennis*	The Netherlands	KP330417	GU237586	GU238046	GU237904
*D. boeremae*	CBS 109942^T^; PD 84/402	*Medicago littoralis* cv. *harbi*	Australia	KT389600	FJ427097	GU238048	FJ426982
*D. brunneospora*	CBS 115.58^T^; FMR 15745	*Chrysanthemum roseum*	Germany	KT389625	KT389802	KT389723	KT389505
*D. calidophila*	CBS 448.83^T^	Desert soil	Egypt	–	FJ427168	GU238052	FJ427059
*D. chenopodii*	CBS 128.93^R^; PD 79/140	*Chenopodium quinoa* cv. *sajana*	Peru	KT389602	GU237591	GU238055	GU237775
*D. chloroguttulata*	CGMCC 3.18351^T^; LC 7435	Air	China	KY742142	KY742299	KY742211	KY742057
*D. coffeae-arabicae*	CBS 123380^T^; PD 84/1013	*Coffea arabica*	Ethiopia	KT389603	FJ427104	GU238005	FJ426993
*D. dactylidis*	CBS 124513^T^; PD 73/1414	*Dactylis glomerata*	USA	–	GU237599	GU238061	GU237766
***D. degraaffiae***	**CBS 144956^T^; JW 195004**	Garden soil	The Netherlands	**MN824470**	**MN824618**	**MN823295**	**MN823444**
*D. dimorpha*	CBS 346.82^T^	*Opuntia phyllocladium*	Spain	–	GU237606	GU238068	GU237835
*D. ellipsoidea*	CGMCC 3.18350^T^; LC 7434	Air	China	KY742145	KY742302	KY742214	KY742060
*D. eucalyptica*	CBS 377.91^R^; PD 79/210	*Eucalyptus* sp.	Australia	KT389605	GU237562	GU238007	GU237846
*D. exigua*	CBS 183.55^T^	*Rumex arifolius*	France	EU874850	GU237525	EU754155	GU237794
*D. gardeniae*	CBS 626.68^T^; IMI 108771	*Gardenia jasminoides*	India	KT389606	FJ427114	GQ387595	FJ427003
*D. glomerata*	CBS 528.66^R^; PD 63/590	*Chrysanthemum* sp.	The Netherlands	GU371781	FJ427124	EU754184	FJ427013
*D. heteroderae*	CBS 109.92^T^; PD 73/1405	Undefined food material	The Netherlands	KT389601	FJ427098	GU238002	FJ426983
*D. ilicicola*	CGMCC 3.18355^T^; LC 8126	*Ilex chinensis*	Italy	KY742150	KY742307	KY742219	KY742065
*D. infuscatispora*	CGMCC 3.18356^T^; LC 8128	*Chrysanthemum indicum*	China	KY742152	KY742309	KY742221	KY742067
*D. keratinophila*	CBS 143032^T^; FMR 13690	Human superficial tissue	USA	LT593039	LT592970	LN907343	LT592901
***D. kooimaniorum***	**CBS 144951^T^; JW 27006**	Garden soil	The Netherlands	**MN824474**	**MN824622**	**MN823299**	**MN823448**
*D. lethalis*	CBS 103.25	Unknown	Unknown	KT389607	GU237564	GU238010	GU237729
*D. macrophylla*	CGMCC 3.18357^T^; LC 8131	*Hydrangea macrophylla*	Italy	KY742154	KY742312	KY742224	KY742070
*D. macrostoma*	JW 57015	Garden soil	The Netherlands	**MN824472**	**MN824620**	**MN823297**	**MN823446**
	CBS 223.69^R^	*Acer pseudoplatanus*	Switzerland	KT389608	GU237623	GU238096	GU237801
	JW 149014	Garden soil	The Netherlands	**MN824473**	**MN824621**	**MN823298**	**MN823447**
	CBS 482.95	*Larix decidua*	Germany	KT389609	GU237626	GU238099	GU237869
*D. maydis*	CBS 588.69^T^	*Zea mays*	USA	GU371782	FJ427190	EU754192	FJ427086
*D. microchlamydospora*	CBS 105.95^T^	*Eucalyptus* sp.	UK	KP330424	FJ427138	GU238104	FJ427028
*D. molleriana*	CBS 229.79^R^	*Digitalis purpurea*	New Zealand	KP330418	GU237605	GU238067	GU237802
*D. negriana*	CBS 358.71^R^	*Vitis vinifera*	Germany	KT389610	GU237635	GU238116	GU237838
*D. nigricans*	CBS 444.81^T^; PDDCC 6546	*Actinidia chinensis*	New Zealand	–	GU237558	GU238000	GU237867
	PD 77/919	*Actinidea chinensis*	Unknown	–	GU237559	GU238001	GU237915
*D. ocimicola*	CGMCC 3.18358^T^; LC 8137	*Ocimum* sp.	China	–	KY742320	KY742232	KY742078
*D. pedeiae*	CBS 124517^T^; PD 92/612A	*Schefflera elegantissima*	The Netherlands	KT389612	GU237642	GU238127	GU237770
*D. pinodella*	LC 8139	*Acer palmatum*	Japan	KY742161	KY742322	KY742234	KY742080
	CBS 531.66	*Trifolium pratense*	USA	KT389613	FJ427162	GU238017	FJ427052
*D. pinodes*	CBS 525.77^T^	*Pisum sativum*	Belgium	KT389614	GU237572	GU238023	GU237883
*D. pomorum*	JW 196022	Garden soil	The Netherlands	**MN824469**	**MN824617**	**MN823294**	**MN823443**
	CBS 539.66^R^; IMI 122266; PD 64/914	*Polygonum tataricum*	The Netherlands	KT389618	FJ427166	GU238028	FJ427056
*D. protuberans*	CBS 381.96^T^; PD 71/706	*Lycium halifolium*	The Netherlands	KT389620	GU237574	GU238029	GU237853
*D. pteridis*	CBS 379.96^T^	*Pteris* sp.	The Netherlands	KT389624	KT389801	KT389722	KT389504
*D. rhei*	CBS 109177^R^; PD 2000/9941	*Rheum rhaponticum*	New Zealand	KP330428	GU237653	GU238139	GU237743
*D. rumicicola*	CBS 683.79^T^	*Rumex obtusifolius*	New Zealand	KT389622	KT389800	KT389721	KT389503
	CBS 179.97	*Rumex hydrolapathum*	The Netherlands	KP330415	GU237575	GU238034	GU237793
	CBS 539.77	*Rumex obtusifolius*	New Zealand	**MN824471**	**MN824619**	**MN823296**	**MN823445**
*D. sancta*	CBS 281.83^T^	*Ailanthus altissima*	South Africa	KT389623	FJ427170	GU238030	FJ427063
*D. segeticola*	CGMCC 3.17489^T^; LC 1636	*Cirsium segetum*	China	KP330414	KP330399	KP330455	KP330443
*D. senecionicola*	CBS 160.78^R^	*Senecio jacobaea*	New Zealand	–	GU237657	GU238143	GU237787
*D. subglomerata*	CBS 110.92^R^; PD 76/1010	*Triticum* sp.	USA	KT389626	FJ427186	GU238032	FJ427080
*D. subherbarum*	CBS 250.92^T^; PD 92/371	*Zea mays*	Canada	–	GU237659	GU238145	GU237809
*D. suiyangensis*	CGMCC 3.18352^T^; LC 7439	Air	China	KY742169	KY742332	KY742244	KY742090
*D. viburnicola*	CBS 523.73^R^; PD 69/800	*Viburnum cassioides*	The Netherlands	KP330430	GU237667	GU238155	GU237879
*Ectophoma multirostrata*	CBS 274.60^T^; FMR 15335; IMI 081598	Soil	India	LT623265	FJ427141	GU238111	FJ427031
*Ec. pomi*	CBS 267.92^T^; FMR 15346; PD 76/1014	*Coffea arabica*	India	LT623263	GU237643	GU238128	GU237814
*Epicoccum nigrum*	CBS 173.73^T^; IMI 164070	*Dactylis glomerata*	USA	KT389632	FJ427107	GU237975	FJ426996
	LC 8157	*Ocimum* sp.	China	KY742179	KY742352	KY742264	KY742110
	LC 5180	*Lonicera japonica*	China	KY742178	KY742351	KY742263	KY742109
	LC 8158	*Poa annua*	USA	KY742180	KY742353	KY742265	KY742111
*Ep. pimprinum*	CBS 246.60^T^; IMI 081601	Soil	India	–	FJ427159	GU237976	FJ427049
	PD 77/1028	Unknown	Unknown	KT389633	FJ427160	GU237977	FJ427050
*Heterophoma sylvatica*	CBS 874.97^T^; PD 93/764	*Melampyrum pratense*	The Netherlands	–	GU237662	GU238148	GU237907
*H. verbascicola*	CGMCC 3.18364^T^; LC 8163	*Verbascum thapsus*	China	KY742187	KY742361	KY742273	KY742119
*Juxtiphoma eupyrena*	JW 164001	Garden soil	The Netherlands	**MN824541**	**MN824689**	**MN823366**	**MN823515**
	JW 263011	Garden soil	The Netherlands	**MN824542**	**MN824690**	**MN823367**	**MN823516**
	JW 158007	Garden soil	The Netherlands	**MN824543**	**MN824691**	**MN823368**	**MN823517**
	JW 201014	Garden soil	The Netherlands	**MN824544**	**MN824692**	**MN823369**	**MN823518**
	JW 213001	Garden soil	The Netherlands	**MN824545**	**MN824693**	**MN823370**	**MN823519**
	JW 201009	Garden soil	The Netherlands	**MN824546**	**MN824694**	**MN823371**	**MN823520**
	JW 4005	Garden soil	The Netherlands	**MN824547**	**MN824695**	**MN823372**	**MN823521**
	JW 4017	Garden soil	The Netherlands	**MN824548**	**MN824696**	**MN823373**	**MN823522**
	JW 3015	Garden soil	The Netherlands	**MN824549**	**MN824697**	**MN823374**	**MN823523**
	JW 224006	Garden soil	The Netherlands	**MN824550**	**MN824698**	**MN823375**	**MN823524**
	JW 132015	Garden soil	The Netherlands	**MN824551**	**MN824699**	**MN823376**	**MN823525**
*Juxtiphoma eupyrena*	JW 146002	Garden soil	The Netherlands	–	**MN824700**	**MN823377**	**MN823526**
	JW 160021	Garden soil	The Netherlands	**MN824552**	**MN824701**	**MN823378**	**MN823527**
	JW 18016	Garden soil	The Netherlands	**MN824553**	**MN824702**	**MN823379**	**MN823528**
	JW 40009	Garden soil	The Netherlands	**MN824554**	**MN824703**	**MN823380**	**MN823529**
	JW 40019	Garden soil	The Netherlands	**MN824555**	**MN824704**	**MN823381**	**MN823530**
	JW 97009	Garden soil	The Netherlands	**MN824556**	**MN824705**	**MN823382**	**MN823531**
	JW 96020	Garden soil	The Netherlands	**MN824557**	**MN824706**	**MN823383**	**MN823532**
	JW 57007	Garden soil	The Netherlands	**MN824558**	**MN824707**	**MN823384**	**MN823533**
	JW 149010	Garden soil	The Netherlands	**MN824559**	**MN824708**	**MN823385**	**MN823534**
	JW 74008	Garden soil	The Netherlands	**MN824560**	**MN824709**	**MN823386**	**MN823535**
	JW 247003	Garden soil	The Netherlands	**MN824561**	**MN824710**	**MN823387**	**MN823536**
	JW 267005	Garden soil	The Netherlands	**MN824562**	**MN824711**	**MN823388**	**MN823537**
	JW 261008	Garden soil	The Netherlands	**MN824563**	**MN824712**	**MN823389**	**MN823538**
	JW 30012	Garden soil	The Netherlands	**MN824564**	**MN824713**	**MN823390**	**MN823539**
	JW 167015	Garden soil	The Netherlands	**MN824565**	**MN824714**	**MN823391**	**MN823540**
	JW 221022B	Garden soil	The Netherlands	**MN824566**	**MN824715**	**MN823392**	**MN823541**
	JW 259004	Garden soil	The Netherlands	**MN824567**	**MN824716**	**MN823393**	**MN823542**
	JW 73004	Garden soil	The Netherlands	**MN824568**	**MN824717**	**MN823394**	**MN823543**
	JW 170018	Garden soil	The Netherlands	**MN824569**	**MN824718**	**MN823395**	**MN823544**
	JW 141018	Garden soil	The Netherlands	**MN824570**	**MN824719**	**MN823396**	**MN823545**
	JW 181003	Garden soil	The Netherlands	**MN824571**	**MN824720**	**MN823397**	**MN823546**
	JW 289013	Garden soil	The Netherlands	**MN824572**	**MN824721**	**MN823398**	**MN823547**
	JW 127004	Garden soil	The Netherlands	**MN824573**	**MN824722**	**MN823399**	**MN823548**
	JW 81007	Garden soil	The Netherlands	**MN824574**	**MN824723**	**MN823400**	**MN823549**
	JW 182002	Garden soil	The Netherlands	**MN824575**	**MN824724**	**MN823401**	**MN823550**
	JW 212001	Garden soil	The Netherlands	**MN824576**	**MN824725**	**MN823402**	**MN823551**
	JW 191036	Garden soil	The Netherlands	**MN824577**	**MN824726**	**MN823403**	**MN823552**
	JW 221020	Garden soil	The Netherlands	**MN824578**	**MN824727**	**MN823404**	**MN823553**
	JW 96002	Garden soil	The Netherlands	**MN824579**	**MN824728**	**MN823405**	**MN823554**
	JW 52011	Garden soil	The Netherlands	**MN824580**	**MN824729**	**MN823406**	**MN823555**
	JW 38012	Garden soil	The Netherlands	**MN824581**	**MN824730**	**MN823407**	**MN823556**
	JW 40007	Garden soil	The Netherlands	**MN824582**	**MN824731**	**MN823408**	**MN823557**
	JW 43007	Garden soil	The Netherlands	**MN824583**	**MN824732**	**MN823409**	**MN823558**
*Juxtiphoma eupyrena*	JW 75002	Garden soil	The Netherlands	**MN824584**	**MN824733**	**MN823410**	**MN823559**
	JW 116017	Garden soil	The Netherlands	**MN824585**	**MN824734**	**MN823411**	**MN823560**
	JW 170013	Garden soil	The Netherlands	**MN824586**	**MN824735**	**MN823412**	**MN823561**
	JW 79016	Garden soil	The Netherlands	**MN824587**	**MN824736**	**MN823413**	**MN823562**
	CBS 374.91; FMR 15329	*Solanum tuberosum*	The Netherlands	LT623268	FJ427110	GU238072	FJ426999
	JW 125024	Garden soil	The Netherlands	**MN824588**	**MN824737**	**MN823414**	**MN823563**
	JW 158014	Garden soil	The Netherlands	**MN824589**	**MN824738**	**MN823415**	**MN823564**
	JW 4010	Garden soil	The Netherlands	**MN824590**	**MN824739**	**MN823416**	**MN823565**
	JW 202020	Garden soil	The Netherlands	**MN824591**	**MN824740**	**MN823417**	**MN823566**
*J. kolkmaniorum*	JW 125028	Garden soil	The Netherlands	**MN824592**	**MN824741**	**MN823418**	**MN823567**
	CBS 146005^T^; JW 185006	Garden soil	The Netherlands	**MN824593**	**MN824742**	**MN823419**	**MN823568**
	JW 191004	Garden soil	The Netherlands	**MN824594**	**MN824743**	**MN823420**	**MN823569**
	JW 23021	Garden soil	The Netherlands	**MN824595**	**MN824744**	**MN823421**	**MN823570**
	JW 167004	Garden soil	The Netherlands	**MN824596**	**MN824745**	**MN823422**	**MN823571**
	JW 221010	Garden soil	The Netherlands	**MN824597**	**MN824746**	**MN823423**	**MN823572**
	JW 220011	Garden soil	The Netherlands	**MN824598**	**MN824747**	**MN823424**	**MN823573**
	JW 241011	Garden soil	The Netherlands	**MN824599**	**MN824748**	**MN823425**	**MN823574**
	JW 94009	Garden soil	The Netherlands	**MN824600**	**MN824749**	**MN823426**	**MN823575**
	CBS 527.66; FMR 15337	Wheat field soil	Germany	LT623269	FJ427111	GU238073	FJ427000
	JW 63001	Garden soil	The Netherlands	**MN824601**	**MN824750**	**MN823427**	**MN823576**
	JW 168007	Garden soil	The Netherlands	**MN824602**	**MN824751**	**MN823428**	**MN823577**
*Leptosphaeria doliolum*	CBS 505.75^T^	*Urtica dioica*	The Netherlands	KT389640	JF740144	GQ387576	JF740205
*Leptosphaerulina australis*	CBS 311.51	Lawn	Switzerland	–	–	FJ795508	–
*L. saccharicola*	CBS 939.69	Soil	The Netherlands	–	GU237541	JX681098	GU237911
*L. trifolii*	CBS 235.58	*Trifolium* sp.	The Netherlands	–	GU237542	GU237982	GU237806
*Macroventuria anomochaeta*	CBS 525.71^T^	Decayed canvas	South Africa	GU456346	GU237544	GU237984	GU237881
*Ma. wentii*	CBS 526.71^T^	Plant litter	USA	KT389642	GU237546	GU237986	GU237884
*Microsphaeropsis olivacea*	CBS 233.77	*Pinus laricio*	France	KT389643	GU237549	GU237988	GU237803
	CBS 442.83	*Taxus baccata*	The Netherlands	–	GU237547	EU754171	GU237865
*Mi. proteae*	CBS 111319^T^; CPC 1425	*Protea nitida*	Unknown	–	JN712650	JN712563	JN712497
*Neoascochyta argentina*	CBS 112524^T^	*Triticum aestivum*	Argentina	–	KT389822	KT389742	KT389524
*Neoa. desmazieri*	CBS 297.69^T^	*Lolium perenne*	Germany	KT389644	KT389806	KT389726	KT389508
*Neoa. paspali*	CBS 560.81^T^; PDDCC 6614	*Paspalum dilatatum*	New Zealand	KP330426	FJ427158	GU238124	FJ427048
*Neoa. tardicrebrescens*	CBS 689.97^T^	Hay	Norway	KT389654	KT389824	KT389744	KT389526
*Neoa. triticicola*	CBS 544.74^T^	*Triticum aestivum*	South Africa	KT389652	GU237488	EU754134	GU237887
*Neodidymelliopsis cannabis*	CBS 234.37	*Cannabis sativa*	Unknown	KP330403	GU237523	GU237961	GU237804
	CBS 121.75^T^; IMI 194767; PD 73/584	*Urtica dioica*	The Netherlands	–	GU237535	GU237972	GU237761
*Neod. polemonii*	CBS 109181^T^; PD 83/757	*Polemonium caeruleum*	The Netherlands	KP330427	GU237648	GU238133	GU237746
*Neod. xanthina*	CBS 383.68^T^	*Delphinium* sp.	The Netherlands	KP330431	GU237668	GU238157	GU237855
*Neomicrosphaeropsis italica*	MFLUCC 16-0284	*Tamarix* sp.	Italy	KU714604	–	KU900296	KU900321
	MFLUCC 15-0484	*Tamarix* sp.	Italy	KU695539	–	KU729853	KU900319
	MFLUCC 15-0485^T^	*Tamarix* sp.	Italy	KU674820	–	KU729854	KU900318
*Nothophoma anigozanthi*	CBS 381.91^T^; FMR 14914	*Anigozanthus maugleisii*	The Netherlands	KT389655	GU237580	GU238039	GU237852
*N. arachidis-hypogaeae*	CBS 125.93^R^; PD 77/1029	*Arachis hypogaea*	India	KT389656	GU237583	GU238043	GU237771
***N. brennandiae***	**JW 1066**	Garden soil	The Netherlands	**MN824603**	**MN824752**	**MN823429**	**MN823578**
	**CBS 145912^T^; JW 53011**	Garden soil	The Netherlands	**MN824604**	**MN824753**	**MN823430**	**MN823579**
	MFLUCC 16-1392	*Ulmus* (×) *hollandica*	Italy	KY053898	KY053899	KY053897	KY053896
*N. gossypiicola*	CBS 377.67; FMR 14912	*Gossypium* sp.	USA	KT389658	GU237611	GU238079	GU237845
	UTHSC:DI16-294	Human deep tissue/ fluids	USA	LT593082	LT593012	LN907437	LT592943
*N. infossa*	CBS 123395^T^	*Fraxinus pennsylvanica*	Argentina	KT389659	FJ427135	GU238089	FJ427025
	CBS 123394	*Fraxinus pennsylvanica*	Argentina	–	FJ427134	GU238088	FJ427024
*N. macrospora*	CBS 140674^T^; FMR 13767	Human respiratory tract	USA	LT593073	LN880539	LN880537	LN880536
*N. pruni*	MFLUCC 18-1600^T^	*Prunus avium*	China	MH853664	MH853671	MH827028	MH827007
*N. quercina*	MFLUCC 18-1588	*Prunus avium*	China	MH853665	MH853672	MH827029	MH827008
	CBS 633.92^R^; ATCC 36786	*Microsphaera alphitoides* from *Quercus* sp.	Ukraine	KT389657	GU237609	EU754127	GU237900
	UTHSC:DI16-270; FMR 13761	Human superficial tissue	USA	LT593067	LT592998	LN907413	LT592929
*N. variabilis*	CBS 142457^T^; FMR 13777	Human respiratory tract	USA	LT593078	LT593008	LN907428	LT592939
*Paraboeremia adianticola*	CBS 260.92; PD 86/1103	*Pteris ensiformis*	Unknown	–	KT389832	KT389752	KT389534
*P. adianticola*	CBS 187.83; PD 82/128; FMR 15344	*Polystichum adiantiforme*	USA	KP330401	GU237576	GU238035	GU237796
*P. camelliae*	CGMCC 3.18108	*Camellia* sp.	China	KX829052	KX829060	KX829044	KX829036
	CGMCC 3.18106^T^	*Camellia* sp.	China	KX829050	KX829058	KX829042	KX829034
	CGMCC 3.18107	*Camellia* sp.	China	KX829051	KX829059	KX829043	KX829035
*P. litseae*	CGMCC 3.18110; LC 5030	*Litsea* sp.	China	KX829046	KX829054	KX829038	KX829030
	JW 157001	Garden soil	The Netherlands	**MN824519**	**MN824667**	**MN823344**	**MN823493**
	CGMCC 3.18109^T^; LC 5028	*Litsea* sp.	China	KX829045	KX829053	KX829037	KX829029
*P. putaminum*	JW 110005	Garden soil	The Netherlands	**MN824480**	**MN824628**	**MN823305**	**MN823454**
	JW 126003	Garden soil	The Netherlands	**MN824481**	**MN824629**	**MN823306**	**MN823455**
	JW 265009	Garden soil	The Netherlands	**MN824482**	**MN824630**	**MN823307**	**MN823456**
	JW 221011	Garden soil	The Netherlands	**MN824483**	**MN824631**	**MN823308**	**MN823457**
	JW 165006	Garden soil	The Netherlands	**MN824484**	**MN824632**	**MN823309**	**MN823458**
	JW 232004	Garden soil	The Netherlands	**MN824485**	**MN824633**	**MN823310**	**MN823459**
	JW 192007	Garden soil	The Netherlands	**MN824486**	**MN824634**	**MN823311**	**MN823460**
	JW 125011	Garden soil	The Netherlands	**MN824487**	**MN824635**	**MN823312**	**MN823461**
	JW 18014	Garden soil	The Netherlands	**MN824488**	**MN824636**	**MN823313**	**MN823462**
	JW 142002	Garden soil	The Netherlands	**MN824489**	**MN824637**	**MN823314**	**MN823463**
	JW 221018	Garden soil	The Netherlands	**MN824490**	**MN824638**	**MN823315**	**MN823464**
	JW 238003	Garden soil	The Netherlands	**MN824491**	**MN824639**	**MN823316**	**MN823465**
	JW 192019	Garden soil	The Netherlands	**MN824492**	**MN824640**	**MN823317**	**MN823466**
	JW 213009	Garden soil	The Netherlands	**MN824493**	**MN824641**	**MN823318**	**MN823467**
	JW 226017	Garden soil	The Netherlands	**MN824494**	**MN824642**	**MN823319**	**MN823468**
	JW 109022	Garden soil	The Netherlands	**MN824495**	**MN824643**	**MN823320**	**MN823469**
	JW 4002	Garden soil	The Netherlands	**MN824496**	**MN824644**	**MN823321**	**MN823470**
	CBS 130.69^R^; IMI 331916	*Malus sylvestris*	Denmark	–	GU237652	GU238138	GU237777
	JW 16015	Garden soil	The Netherlands	**MN824497**	**MN824645**	**MN823322**	**MN823471**
	JW 16001	Garden soil	The Netherlands	**MN824498**	**MN824646**	**MN823323**	**MN823472**
	JW 25002	Garden soil	The Netherlands	**MN824499**	**MN824647**	**MN823324**	**MN823473**
	JW 276009	Garden soil	The Netherlands	**MN824500**	**MN824648**	**MN823325**	**MN823474**
	JW 48011	Garden soil	The Netherlands	**MN824501**	**MN824649**	**MN823326**	**MN823475**
	JW 4011	Garden soil	The Netherlands	**MN824502**	**MN824650**	**MN823327**	**MN823476**
	JW 276008	Garden soil	The Netherlands	**MN824503**	**MN824651**	**MN823328**	**MN823477**
	JW 65008	Garden soil	The Netherlands	**MN824505**	**MN824653**	**MN823330**	**MN823479**
	JW 132016	Garden soil	The Netherlands	**MN824506**	**MN824654**	**MN823331**	**MN823480**
	JW 226014	Garden soil	The Netherlands	**MN824507**	**MN824655**	**MN823332**	**MN823481**
	JW 226015	Garden soil	The Netherlands	**MN824508**	**MN824656**	**MN823333**	**MN823482**
	JW 25012	Garden soil	The Netherlands	**MN824509**	**MN824657**	**MN823334**	**MN823483**
*P. putaminum*	JW 11007	Garden soil	The Netherlands	**MN824510**	**MN824658**	**MN823335**	**MN823484**
	JW 129005	Garden soil	The Netherlands	**MN824511**	**MN824659**	**MN823336**	**MN823485**
	CBS 372.91^R^; PD 75/690	*Ceratocystis ulmi*	The Netherlands	–	GU237651	GU238137	GU237843
	JW 145026	Garden soil	The Netherlands	**MN824504**	**MN824652**	**MN823329**	**MN823478**
	JW 4006	Garden soil	The Netherlands	**MN824512**	**MN824660**	**MN823337**	**MN823486**
	JW 191017	Garden soil	The Netherlands	**MN824513**	**MN824661**	**MN823338**	**MN823487**
	JW 161002	Garden soil	The Netherlands	**MN824514**	**MN824662**	**MN823339**	**MN823488**
	JW 116031	Garden soil	The Netherlands	**MN824515**	**MN824663**	**MN823340**	**MN823489**
	JW 1008	Garden soil	The Netherlands	**MN824516**	**MN824664**	**MN823341**	**MN823490**
	JW 1020	Garden soil	The Netherlands	**MN824517**	**MN824665**	**MN823342**	**MN823491**
	JW 1046	Garden soil	The Netherlands	**MN824518**	**MN824666**	**MN823343**	**MN823492**
***P. rekkeri***	**JW 13016**	Garden soil	The Netherlands	**MN824526**	**MN824674**	**MN823351**	**MN823500**
	**JW 13030**	Garden soil	The Netherlands	**MN824527**	**MN824675**	**MN823352**	**MN823501**
	**JW 79024**	Garden soil	The Netherlands	**MN824528**	**MN824676**	**MN823353**	**MN823502**
	**JW 25013**	Garden soil	The Netherlands	**MN824529**	**MN824677**	**MN823354**	**MN823503**
	**JW 167006**	Garden soil	The Netherlands	**MN824530**	**MN824678**	**MN823355**	**MN823504**
	**JW 132004**	Garden soil	The Netherlands	**MN824531**	**MN824679**	**MN823356**	**MN823505**
	**CBS 144949; JW 4024**	Garden soil	The Netherlands	**MN824532**	**MN824680**	**MN823357**	**MN823506**
	**JW 13017**	Garden soil	The Netherlands	**MN824533**	**MN824681**	**MN823358**	**MN823507**
	**JW 91008**	Garden soil	The Netherlands	**MN824534**	**MN824682**	**MN823359**	**MN823508**
	**JW 226002**	Garden soil	The Netherlands	**MN824535**	**MN824683**	**MN823360**	**MN823509**
	**JW 3018**	Garden soil	The Netherlands	**MN824536**	**MN824684**	**MN823361**	**MN823510**
	**CBS 144955^T^; JW 172002**	Garden soil	The Netherlands	**MN824537**	**MN824685**	**MN823362**	**MN823511**
	**JW 51014**	Garden soil	The Netherlands	**MN824538**	**MN824686**	**MN823363**	**MN823512**
	**JW 196020**	Garden soil	The Netherlands	**MN824539**	**MN824687**	**MN823364**	**MN823513**
	**CBS 144950; JW 6005**	Garden soil	The Netherlands	**MN824540**	**MN824688**	**MN823365**	**MN823514**
*P. selaginellae*	CBS 122.93^T^; PD 77/1049	*Selaginella* sp.	The Netherlands	–	GU237656	GU238142	GU237762
***P. truiniorum***	**JW 270002**	Garden soil	The Netherlands	**MN824520**	**MN824668**	**MN823345**	**MN823494**
	**CBS 144952^T^; JW 47002**	Garden soil	The Netherlands	**MN824521**	**MN824669**	**MN823346**	**MN823495**
	**JW 147025**	Garden soil	The Netherlands	**MN824522**	**MN824670**	**MN823347**	**MN823496**
	**JW 182014**	Garden soil	The Netherlands	**MN824523**	**MN824671**	**MN823348**	**MN823497**
	**JW 192003**	Garden soil	The Netherlands	**MN824524**	**MN824672**	**MN823349**	**MN823498**
	**CBS 144961; JW 203021**	Garden soil	The Netherlands	**MN824525**	**MN824673**	**MN823350**	**MN823499**
*Phoma herbarum*	CBS 274.37	*Picea excelsa*	UK	KT389662	KT389835	KT389754	KT389537
	CBS 615.75^R^; IMI 199779; PD 73/655	*Rosa multiflora* cv. *cathayensis*	The Netherlands	KP330420	FJ427133	EU754186	FJ427022
*Phomatodes aubrietiae*	CBS 627.97^T^; PD 70/714	*Aubrietia* sp.	The Netherlands	KT389665	GU237585	GU238045	GU237895
*Phomat. nebulosa*	JW 166004	Garden soil	The Netherlands	**MN824609**	**MN824758**	**MN823435**	**MN823584**
	JW 166006	Garden soil	The Netherlands	**MN824610**	**MN824759**	**MN823436**	**MN823585**
	JW 166013	Garden soil	The Netherlands	**MN824611**	**MN824760**	**MN823437**	**MN823586**
	CBS 100191	*Thlapsi arvense*	Poland	KT389666	KP330390	KP330446	KP330434
	CBS 117.93; PD 83/90	*Mercurialis perennis*	The Netherlands	KP330425	GU237633	GU238114	GU237757
*Pseudoascochyta novae-zelandiae*	CBS 141689^T^; FMR 15110	*Cordyline australis*	New Zealand	LT592895	LT592894	LT592893	LT592892
*Pse. pratensis*	CBS 141688^T^; FMR 14524	Soil	Spain	LT223133	LT223132	LT223131	LT223130
*Remotididymella anthropophylica*	CBS 142462^T^; FMR 13770	Human respiratory tract	USA	LT593075	LT593005	LN907421	LT592936
*R. destructiva*	CBS 378.73^T^; FMR 15328	*Lycopersicon esculentum*	Tonga	LT623258	GU237601	GU238063	GU237849
*Stagonosporopsis andigena*	CBS 269.80; PD 75/914	*Solanum* sp.	Peru	–	GU237675	GU238170	GU237817
*S. astragali*	CBS 178.25^R^; MUCL 9915	*Astragalus* sp.	Unknown	–	GU237677	GU238172	GU237792
*S. bomiensis*	LC 8168	Boraginaceae	China	KY742190	KY742366	KY742278	KY742124
	CGMCC 3.18366^T^; LC 8167	Boraginaceae	China	KY742189	KY742365	KY742277	KY742123
*S. crystalliniformis*	CBS 713.85^T^; ATCC 76027; PD 83/826	*Lycopersicon esculentum*	Colombia	KT389675	GU237683	GU238178	GU237903
*S. dorenboschii*	CBS 426.90^T^; IMI 386093; PD 86/551	*Physostegia virginiana*	The Netherlands	KT389678	GU237690	GU238185	GU237862
*S. hortensis*	CBS 104.42^R^	–	The Netherlands	KT389680	GU237703	GU238198	GU237730
	CBS 572.85; PD 79/269	*Phaseolus vulgaris*	The Netherlands	KT389681	GU237704	GU238199	GU237893
*S. loticola*	CBS 562.81^T^; PDDCC 6884	*Lotus pedunculatus*	New Zealand	KT389684	GU237697	GU238192	GU237890
*S. papillata*	LC 8170	*Rumex nepalensis*	China	KY742192	KY742368	KY742280	KY742126
	CGMCC 3.18367^T^; LC 8169	*Rumex nepalensis*	China	KY742191	KY742367	KY742279	KY742125
***S. stuijvenbergii***	**CBS 144953^T^; JW 132011**	Garden soil	The Netherlands	**MN824475**	**MN824623**	**MN823300**	**MN823449**
	**JW 33021**	Garden soil	The Netherlands	**MN824476**	**MN824624**	**MN823301**	**MN823450**
	**JW 14003**	Garden soil	The Netherlands	**MN824477**	**MN824625**	**MN823302**	**MN823451**
	**JW 44014**	Garden soil	The Netherlands	**MN824478**	**MN824626**	**MN823303**	**MN823452**
***S. weymaniae***	**CBS 144959^T^; JW 201003**	Garden soil	The Netherlands	**MN824479**	**MN824627**	**MN823304**	**MN823453**
*Vacuiphoma bulgarica*	CBS 357.84^T^	*Trachystemon orientale*	Bulgaria	LT623256	GU237589	GU238050	GU237837
*Vac. oculihominis*	UTHSC:DI16-308^T^; FMR 13801	Human superficial tissue	USA	LT593093	LT593023	LN907451	LT592954
***Vandijckomycella joseae***	**CBS 144948; JW 1068**	Garden soil	The Netherlands	**MN824614**	**MN824763**	**MN823440**	**MN823589**
***Van. joseae***	**CBS 143011^T^; JW 1073**	Garden soil	The Netherlands	**MN824615**	**MN824764**	**MN823441**	**MN823590**
***Van. snoekiae***	**CBS 144954^T^; JW 149017**	Garden soil	The Netherlands	**MN824616**	**MN824765**	**MN823442**	**MN823591**
*Xenodidymella applanata*	CBS 115577	*Rubus idaeus*	Sweden	KT389688	KT389850	KT389762	KT389546
	CBS 195.36^T^	*Rubus idaeus*	The Netherlands	–	KT389852	KT389764	KT389548
	CBS 205.63	*Rubus idaeus*	The Netherlands	KP330402	GU237556	GU237998	GU237798
	CBS 115578	*Rubus arcticus* nothossp. *stellarcticus*	Sweden	–	KT389851	KT389763	KT389547
*X. asphodeli*	CBS 375.62^T^	*Asphodelus albus*	France	KT389689	KT389853	KT389765	KT389549
	CBS 499.72	*Asphodelus ramosus*	Italy	–	KT389853	KT389766	KT389550
*X. catariae*	CBS 102635; PD 77/1131	*Nepeta catenaria*	The Netherlands	KP330404	GU237524	GU237962	GU237727
*X. humicola*	CBS 220.85^R^; PD 71/1030	*Franseria* sp.	USA	KP330422	GU237617	GU238086	GU237800
***X. weymaniae***	**CBS 144960^T^; JW 201005**	Garden soil	The Netherlands	**MN824613**	**MN824762**	**MN823439**	**MN823588**

^1^ New species are marked in **bold**. ^2^ATCC = American Type Culture Collection, Virginia, USA; CBS = Westerdijk Fungal Biodiversity Institute, Utrecht, The Netherlands; CGMCC = China General Microbiological Culture Collection, Beijing, China; CPC = Culture collection of Pedro Crous, housed at the Westerdijk Fungal Biodiversity Institute, Utrecht, The Netherlands; FMR = Facultat de Medicina, Universitat Rovira i Virgili, Reus, Spain; JW = Johanna Westerdijk working collection housed at the Westerdijk Fungal Biodiversity Institute, Utrecht, The Netherlands; LC = Personal culture collection of Lei Cai, housed at CAS, China; MFLUCC = Mae Fah Luang University Culture Collection, Chiang Rai, Thailand; PD = Plant Protection Service, Wageningen, the Netherlands; PDDCC = Plant Diseases Division Culture Collection, Auckland, New Zealand; UTHSC = Fungus Testing Laboratory at the University of Texas Health Science Center, San Antonio, Texas, USA. ^T^ and ^R^ indicate ex-type and representative strains, respectively. ^3^*rpb2*: partial RNA polymerase II second largest subunit gene; *tub2*: partial β-tubulin gene; ITS: internal transcribed spacers and intervening 5.8S nrDNA; LSU: partial large subunit nrDNA. Strains representing new species are marked in **bold**. Sequences generated in this study are marked in **bold**.

### Selection of Didymellaceae strains

A preliminary species identification of the strains was carried-out by a BLASTn search performed with each ITS and/or LSU sequence against the NCBI (http://blast.ncbi.nlm.nih.gov) and WI (http://www.westerdijkinstitute.nl/Collections) databases. The ITS and/or LSU sequences generated in this study with more than 98 % similarity with reference sequences for Didymellaceae were selected for further study (Table 1).

### Sequence alignment and molecular phylogenetic analysis

To further study the phylogenetic relationships, reference sequences of Didymellaceae were downloaded from GenBank (Table 1). Sequences of single loci were aligned with MAFFT v.7 using default settings (Katoh et al. 2017), and manually edited in MEGA v.6.0 when necessary (Tamura et al. 2013). MrModeltest v.2.3 (Nylander 2004) was used to select the best-fit models of evolution for the four data partitions according to the Akaike information criterion. Bayesian inference (BI), maximum-likelihood (ML) and maximum parsimony (MP) methods were implemented for phylogenetic analysis of individual gene regions and the combined dataset. The multi-locus gene dataset was generated using SequenceMatrix v.1.8 (Vaidya et al. 2011).

Bayesian analyses were performed using MrBayes v.3.2.6 (Ronquist et al. 2012) as described by Chen et al. (2015). The burn-in fraction was set to 0.25, after which the 50 % majority rule consensus trees and posterior probability (PP) values were calculated. The ML analyses including 1000 bootstrap replicates were conducted using RAxML v.7.2.6 (Stamatakis and Alachiotis et al. 2010) as described by Chen et al. (2015). Statistical support for the branches was evaluated using a bootstrap analysis (BS) of 1000 replicates. MP analyses were carried out using PAUP v.4.0b10 (Swofford 2003) as described by Braun et al. (2018). Statistical support for the branches was evaluated using a bootstrap analysis (PBS) of 1000 replicates. Trees were visualised in FigTree v.1.4.0 (Rambaut 2014) and the layout was created in Adobe Illustrator. Alignments and phylogenetic trees derived from this study were uploaded to TreeBASE (www.treebase.org) and sequences deposited in GenBank (Table 1).

### Morphological characterisation

Isolates of Didymellaceae were transferred to fresh oatmeal agar (OA), 2 % malt extract agar (MEA) and potato dextrose agar (PDA) (Crous et al. 2019) plates and incubated at 25 °C under near-ultraviolet (UV) light (12 h light/12 h dark) to induce sporulation. Colony diameters were measured after 7 d of incubation (Boerema et al. 2004), and macroscopic characters and colony colours were described after 14 days of incubation and rated according to the colour charts of Rayner (1970). Preparations were mounted in distilled water to study the micro-morphological structures of mature conidiomata, conidiogenous cells and conidia from OA cultures (Aveskamp et al. 2010; Chen et al. 2015). Morphological observations included the general characteristics of the conidiomata, shape, presence of mycelium/setae on conidiomata, number of ostioles, thickness and texture of the pycnidial wall, length and width of the conidiogenous cells and conidia. To study the pycnidial wall, sections of mature conidiomata were generated using a Leica CM 1900 freezing microtome (Aveskamp et al. 2010; Chen et al. 2015). Observations of micro-morphological characteristics were processed with a Nikon Eclipse 80i compound microscope with differential interference contrast (DIC) optics and a Nikon AZ100 dissecting microscope, both equipped with a Nikon DS-Ri2 high-definition colour digital camera (Nikon, Tokyo, Japan) using NIS-elements imaging software v.4.3. The NaOH spot test was carried out using a drop of concentrated NaOH to determine the secretion of metabolite E on OA cultures (Boerema et al. 2004). Morphological descriptions and taxonomic information for the new taxa were deposited in MycoBank (Crous et al. 2004).

## Results

A total of 293 soil samples were analysed, and nearly 3000 fungal strains were obtained. Among them, 148 Didymellaceae isolates were identified from 89 different garden soil samples, representing several locations in the Netherlands (Table 1).

### Phylogenetic identification

**Figure 1.**
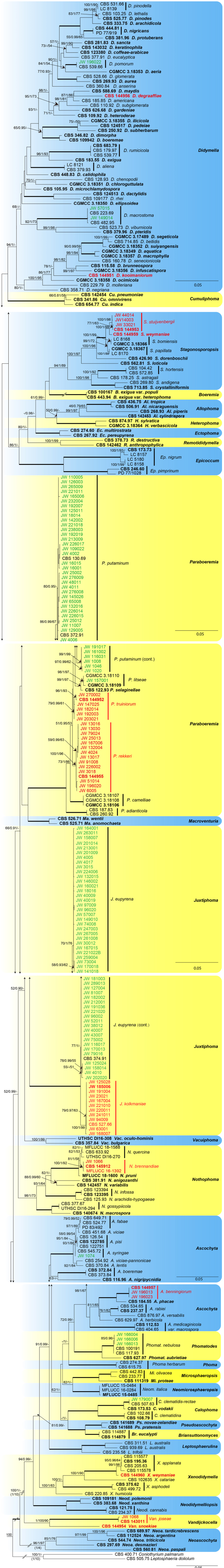
Phylogenetic tree generated from the maximum-likelihood analysis based on the combined ITS, LSU, *tub2* and *rpb2* sequence alignment of Didymellaceae members. The RAxML bootstrap support values (BS), Bayesian posterior probabilities (PP), and parsimony bootstrap support values (PBS) are given at the nodes (BS/PP/PBS). BS and PBS values represent parsimony bootstrap support values >50 %. Full supported branches are indicated in bold. The scale bar represents the expected number of changes per site. Ex-type strains are represented in bold. Strains obtained in the current study are printed in green; among them, whilst strains that represent new taxa are printed in red. Some of the basal branches were shortened to facilitate layout (the fraction in round parentheses refers to the presented length compared to the actual length of the branch). The tree was rooted to *Coniothyrium palmarum* CBS 400.71 and *Leptosphaeria doliolum* CBS 505.75.

A multi-locus phylogeny comprising 325 strains, including the JW soil isolates and reference strains from GenBank, was used to infer the relationships among species in Didymellaceae (Figure 1, Table 1). *Coniothyrium palmarum* (CBS 400.71) and *Leptosphaeria doliolum* (CBS 505.75) were used as outgroups. The final combined ITS, LSU, *rpb2* and *tub2* alignment comprised 2317 characters including gaps (500 for ITS; 859 for LSU; 602 for *rpb2*; 356 for *tub2*), of which 1563 characters were constant, 106 parsimony-uninformative, and 618 were parsimony-informative. For the Bayesian analysis, SYM+I+G was selected as the best-fit model for the ITS dataset, and GTR+I+G was selected as the best model for the LSU, *tub2* and *rpb2* datasets. The phylogenetic trees obtained with three analyses showed a similar topology and were congruent with each other, and only the ML tree is presented herein with BS, PP, and PBS values plotted on the branches (Figure 1).

In the phylogenetic analysis, the 148 isolates from Dutch soil were distributed in 10 clades (Figure 1). The majority of the isolates clustered in *Juxtiphoma* (n=63) which were recovered from 48 soil samples and 28 cities, followed by *Paraboeremia* (n=61) from 29 soil samples and 19 cities. Other isolates belonged to *Didymella* spp. (n=5), *Stagonosporopsis* spp. (n=5), *Ascochyta* spp. (n=4), *Phomatodes nebulosa* (n=3), *Nothophoma* spp. (n=2), *Calophoma clematidis* (n=1), and *Xenodidymella applanata* (n=1), and three isolates clustered in an unknown clade (Figure 1, Table 1).

In the *Juxtiphoma* clade species clustered in two lineages, one corresponding to *J. eupyrena* (77/1/-) and the other representing a potentially new species (100/1/99). In the *Paraboeremia* clade, the soil isolates clustered in *P. putaminum* (86/0.99/67) and *P. litseae* (98/1/97). However, 21 isolates were distributed in two different lineages (with 6 and 15 isolates, respectively) that were phylogenetically distant from other species, representing two potentially new taxa. The soil isolates belonging to *Stagonosporopsis* clustered in a clade (100/1/99) that was phylogenetically distant from the other species, representing two potentially new species. In *Didymella*, the species were distributed in *D. macrostoma* (100/1/100) and *D. pomorum* (100/1/100), while isolates JW 195004 and JW 27006 were placed in two different branches, representing two putative new species. In *Ascochyta* one isolate grouped with *A. syringae* (93/1/86), whereas three isolates grouped in a different clade distant from previously known species, representing a potentially new species (100/1/100). The other three isolates grouped together at the bottom of the tree in a distant unknown lineage, which is introduced herein as a new genus with two species (100/1/90). All the new taxa are introduced in the taxonomy section based on the phylogenetic analysis and supported by morphological data. Descriptions and illustrations of the new taxa are provided in the taxonomy section below.

### Loci resolution

The single locus phylogenies of *rpb2* and *tub2* performed quite well at both generic and species levels. The *rpb2* phylogeny was able to discriminate all 27 generic clades included in the phylogeny (Figure 1), with good resolution of species among these genera (140 of 143 species). The *tub2* phylogeny was able to distinguish 26 of 27 generic clades recognising 134 of 143 species, but proved unsuccessful for *Vacuiphoma* and *Ascochyta*, mainly because species of these genera did not cluster into monophyletic lineages, but were sometimes intermixed or formed separate lineages. However, the LSU phylogeny displayed a low resolution at both generic and species levels, being able to distinguish only 12 of 27 genera and 50 of 143 species. The ITS phylogeny was able to distinguish 17 of the 27 generic clades and 44 of the 143 species.

### Taxonomy

#### Ascochyta benningiorum

Taxon classificationFungiPleosporalesDidymellaceae

Hern.-Restr., L. W. Hou, L. Cai & Crous sp. nov.

79C53355-2002-5B6C-B44A-5142F644BAA9

833194

Figure 2

##### Etymology.

*benningiorum* refers to Eva, Bas & Anne Benning who collected the soil sample from which the ex-type strain was isolated.

##### Typus.

The Netherlands. Gelderland province, Wijchen, isolated from garden soil, Mar. 2017, E. Benning, B. Benning & A. Benning (***holotype*** designated here CBS H-24104, living ex-type culture CBS 144957 = JW 196005).

*Conidiomata* pycnidial, mostly solitary, sometimes confluent, globose or subglobose, irregularly-shaped with age, brown to dark brown, glabrous, mostly produced on the agar surface and some immersed, 140–480(–580) × 100–370(–440) μm; with 1–6(–10) slightly papillate ostioles; pycnidial wall pseudoparenchymatous, 4–8 layers, 14.5–65 μm thick, outer layers composed of brown, flattened polygonal cells of 11–28 μm diam. *Conidiogenous cells* phialidic, hyaline, smooth, globose, ampulliform to lageniform, 5.5–9 × 4–6.5 μm. *Conidia* cylindrical, hyaline, smooth- and thin-walled, mostly straight, occasionally curved, aseptate, (3.5–)4.5–7 × 1.5–2.5 μm, 2-guttulate, small. *Conidia matrix* whitish.

##### Culture characteristics.

Colonies after 7 d at 25 °C, on OA reaching 50–55 mm diam, aerial mycelium floccose, olivaceous to olivaceous black, buff towards the periphery, abundant production of pycnidia, margin irregular; reverse concolorous with the surface. On MEA reaching 40–45 mm diam, aerial mycelium floccose, concentric circles, centre pink, grey olivaceous, mouse grey, rosy buff toward periphery, moderate production of pycnidia, margin irregular; reverse orange, olivaceous black toward periphery. On PDA reaching 45–50 mm diam, aerial mycelium floccose, dark brick to olivaceous grey, buff towards periphery, abundant production of pycnidia, margin irregular; reverse concolorous with the surface. NaOH spot test negative on OA.

##### Additional specimens examined.

The Netherlands. Gelderland province, Wijchen, isolated from garden soil, Mar. 2017, E. Benning, B. Benning & A. Benning, JW 196023 = CBS 144958; ibid. JW 196013.

##### Notes.

*Ascochyta benningiorum* is represented in the phylogenetic tree by three isolates (CBS 144957, CBS 144958 and JW 196013) from the same soil sample collected in Wijchen (Gelderland province). *Ascochyta benningiorum* grouped in a distinct clade close to *A. phacae* (Figure 1). However, it morphologically differs from *A. phacae* by having smaller (3.5–7 × 1.5–2.5 μm) and aseptate conidia. In *A. phacae* the conidia are 7–10 × 2–4 μm and 0–1-septate (Corbaz 1955).

Species in *Ascochyta* are commonly regarded as plant pathogens, especially of cereal crops and legumes (Davidson and Kimber 2007; Tivoli and Banniza 2007), and only a few species were reported from soil, namely *A. fabae*, *A. lentis*, *A. pisi*, *A. rabiei* (Gossen and Morrall 1986; Tivoli and Banniza 2007) and *A. syringae* in the current study. Nevertheless, *A. benningiorum* is phylogenetically and morphologically distinct from these species (Figure 1; Chen et al. 2015).

**Figure 2.**
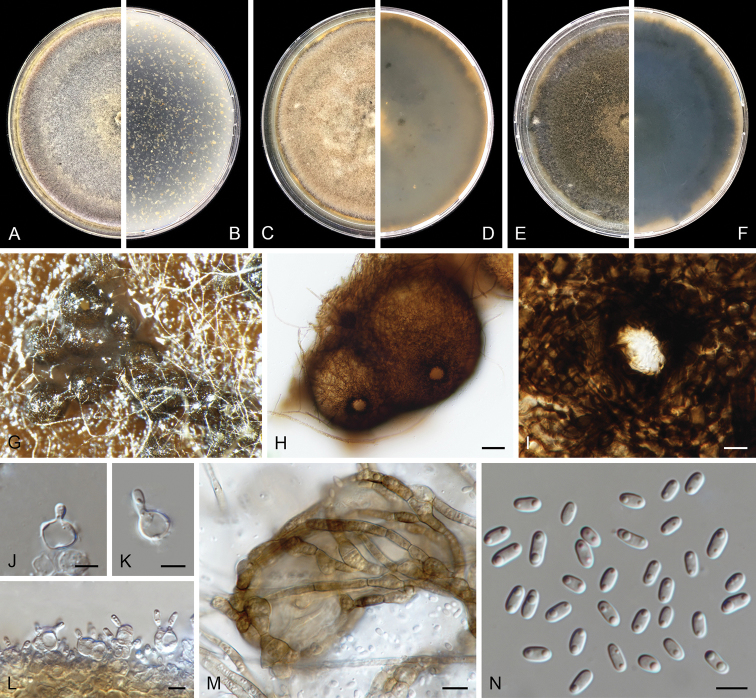
*Ascochyta benningiorum* (CBS 144957). **A, B** Colony on OA (front and reverse) **C, D** colony on MEA (front and reverse) **E, F** colony on PDA (front and reverse) **G** pycnidia forming on OA**H** pycnidium **I** section of pycnidium **J** section of pycnidial wall **K–M** conidiogenous cells **N** conidia. Scale bars: 100 μm (**H, I**); 10 μm (**J**); 5 μm (**K–N**).

#### Didymella degraaffiae

Taxon classificationFungiPleosporalesDidymellaceae

Hern.-Restr., L. W. Hou, L. Cai & Crous sp. nov.

34012AEA-3742-51D5-A477-323A0BB8B57B

833195

Figure 3

##### Etymology.

*degraaffiae* refers to Janne de Graaff who collected the soil sample from which the ex-type strain was isolated.

##### Typus.

The Netherlands. Limburg province, Weert, isolated from garden soil, Mar. 2017, J. de Graaff (***holotype*** designated here CBS H-24105, living ex-type culture CBS 144956 = JW 195004).

*Conidiomata* pycnidial, superficial on the agar or semi-immersed in the agar, scattered or aggregated, mostly confluent, globose, subglobose, lageniform to irregularly-shaped with age, brown to dark brown, ostiolate, covered by hyphal outgrowths, especially near the ostiole, 150–485 × 120–330 μm; non-papillate or with up to two papillate ostioles; pycnidial wall pseudoparenchymatous, 3–6 layers, 10–55 μm thick, outer layers composed of brown, isodiametric cells, 16–33 μm diam. *Conidiogenous cells* phialidic, hyaline, smooth, ampulliform, lageniform, pyriform or globose, 5.5–8.5 × 5–8 μm. *Conidia* ellipsoidal, oblong or oval, thin- and smooth-walled, hyaline, aseptate, 4.5–9(–11) × 3–4.5 μm, 2–6-guttulate, small. *Conidial matrix* milky white.

##### Culture characteristics.

Colonies after 7 d at 25 °C, on OA reaching 65–70 mm diam, aerial mycelium floccose, orange to olivaceous, margin regular; reverse black near the centre, pale grey towards the periphery. On MEA reaching 55–60 mm diam, aerial mycelium floccose, buff to pale olivaceous, with white mycelium pellet and radially furrowed zones near the centre, margin regular; reverse buff near the centre, olivaceous to yellow towards the periphery. On PDA reaching 50–55 mm diam, aerial mycelium floccose, concentric circles pale brown, pale olivaceous grey, dark olivaceous, honey, margin irregular; reverse black with a pale olivaceous edge. NaOH spot test negative on OA.

##### Notes.

In our phylogenetic analysis, *D. degraaffiae* grouped with *D. americana* and *D. maydis* (Figure 1). However, morphologically, *D. americana* differs by its smaller conidiogenous cells (3–5 × 3–4 μm) and conidia (5–7 × 2–2.5 μm) (Boerema 1993); while *D. maydis* differs in having larger conidia (15–17 × 3.5–5 μm) (de Gruyter 2002). Furthermore, *D. americana* and *D. maydis* occasionally produced 1-septate conidia, while septate conidia were not observed in *D. degraaffiae*.

**Figure 3.**
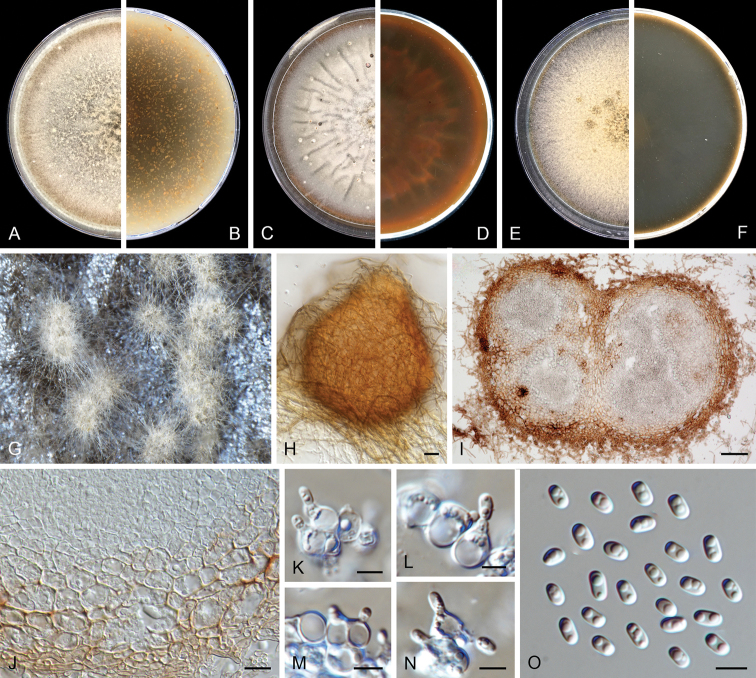
*Didymella degraaffiae* (CBS 144956). **A, B** Colony on OA (front and reverse) **C, D** colony on MEA (front and reverse) **E, F** colony on PDA (front and reverse) **G, H** pycnidia on OA**I** section of pycnidium **J** section of pycnidial wall **K, L** conidiogenous cells **M** chlamydospores **N** conidia. Scale bars: 50 μm (**H, I**); 10 μm (**J**); 5 μm (**K–N**).

#### Didymella kooimaniorum

Taxon classificationFungiPleosporalesDidymellaceae

Hern.-Restr., L. W. Hou, L. Cai & Crous sp. nov.

667CE759-730C-5AB7-8C08-7077CFF0CB63

833196

Figure 4

##### Etymology.

*kooimaniorum* refers to Noud & Robin Kooiman who collected the soil sample from which the ex-type strain was isolated.

##### Typus.

The Netherlands. Utrecht province, Vleuten, isolated from garden soil, Mar. 2017, N. Kooiman & R. Kooiman (***holotype*** designated here CBS H-24106, living ex-type culture CBS 144951 = JW 27006).

*Conidiomata* pycnidial, superficial or semi-immersed, scattered or solitary, sometimes confluent, globose to subglobose, irregularly-shaped with age, pale brown to brown, covered by hyphal outgrowths, especially near the ostioles, 200–375 × 195–280 μm; with 1–3(–6) papillate ostioles; pycnidial wall pseudoparenchymatous, 3–5 layers, 10–35 μm thick, outer layers composed of pale brown, flattened polygonal cells of 16–32 μm diam. *Conidiogenous cells* phialidic, hyaline, smooth, ampulliform, lageniform or somewhat isodiametric, (4.5–)5.5–10 × 3.5–9 μm. *Conidia* ellipsoidal to oblong, straight, thin- and smooth-walled, hyaline, aseptate, 3.5–7 × 2–3 μm, 2-guttulate, big. *Conidial matrix* buff.

##### Culture characteristics.

Colonies after 7 d at 25 °C, on OA reaching 55–60 mm diam, aerial mycelium floccose, pale smoke grey, pale brown towards periphery, abundant production of confluent pycnidia, margin regular; reverse pale olivaceous, with some olivaceous black zones. On MEA reaching 50–55 mm diam, aerial mycelium woolly, pale olivaceous grey, margin irregular; reverse buff near the centre, dark brown with orange edge. On PDA reaching 50–55 mm diam, aerial mycelium floccose, pale mouse grey with olivaceous edge, margin irregular; reverse dark brown with pale brown edge. NaOH spot test negative on OA.

##### Notes.

Based on the multi-gene phylogenetic analyses, *D. kooimaniorum* forms an independent branch, clearly separated from other species in *Didymella* (Figure 1). Morphologically, *D. kooimaniorum* is characterised by pale brown pycnidia densely covered by long hairs, and ostioles with up to six papillae with a darker neck.

**Figure 4.**
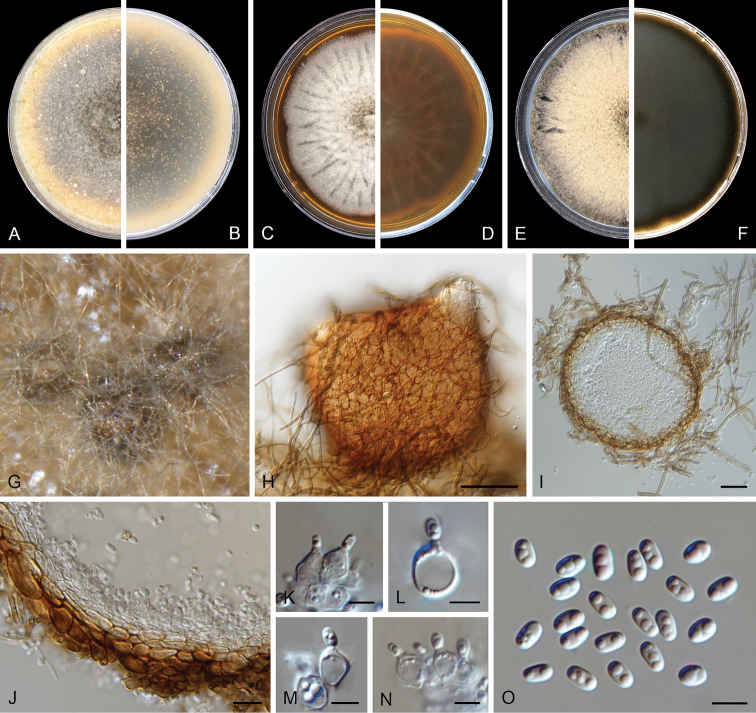
*Didymella kooimaniorum* (CBS 144951). **A, B** Colony on OA (front and reverse) **C, D** colony on MEA (front and reverse) **E, F** colony on PDA (front and reverse) **G** pycnidia forming on OA**H** pycnidia **I** section of pycnidium **J** section of pycnidial wall **K–M** conidiogenous cells **N** conidia. Scale bars: 100 μm (**H**); 50 μm (**I**); 10 μm (**J**); 5 μm (**K–N**).

#### Juxtiphoma kolkmaniorum

Taxon classificationFungiPleosporalesDidymellaceae

Hern.-Restr., L. W. Hou, L. Cai & Crous sp. nov.

329B1DDF-FA2D-5C9F-AE49-B48ABAA4553D

833197

Figure 5

##### Etymology.

*kolkmaniorum* refers to Linde & Mette Kolkman who collected the soil sample from which the ex-type strain was isolated.

##### Typus.

The Netherlands. Ophemert, isolated from garden soil, Mar. 2017, L. & M. Kolkman (***holotype*** designated here CBS H-24214, living ex-type culture CBS 146005 = JW 185006).

*Conidiomata* pycnidial, superficial, solitary or confluent, globose to subglobose, brown to dark brown, glabrous, covered by dark hyphae and chlamydospores, 100–350 μm; uniostiolate papillate; pycnidial wall pseudoparenchymatous, 2–4 layers, 7.5–12.5 μm thick, outer layer composed of brown, flattened polygonal cells. *Conidiogenous cells* mono- or polyphialidic, hyaline, smooth, subcylindrical, ampulliform or somewhat isodiametric, 5.5–11.5 × 2.5–5.5 μm. *Conidia* ellipsoidal to oblong, straight or curved, thin- and smooth-walled, hyaline, aseptate, 3.5–7.5 × 2–3 μm, 1–3-guttulate, medium. *Conidial matrix* white to buff. *Chlamydospores* terminal or intercalary, solitary, or in simple or branched chains, barrel-shaped, subglobose or ellipsoidal, pale brown to brown, guttulate, 5.5–12 × 4–8 μm.

##### Culture characteristics.

Colonies after 7 d at 25 °C, on OA reaching 45–60 mm diam, aerial mycelium cottony to floccose, isabelline to olivaceous, margin irregular; reverse concolorous. On MEA reaching 45–55 mm diam, aerial mycelium cottony to floccose, smoke grey to pale olivaceous grey with white edge, margin entire; reverse buff to smoke grey near the centre, olivaceous black with buff edge. On PDA reaching 45–50 mm diam, aerial mycelium cottony to floccose, olivaceous buff, dull green to buff, margin irregular; reverse smoke grey near the centre, olivaceous black with buff edge. NaOH spot test negative on OA.

##### Additional specimens examined.

Germany. Kiel-Kitzeberg, from wheat field soil, 1966, W. Gams, living cultures CBS 527.66 = FMR 15337 = ATCC 22238; The Netherlands. North Brabant province, Breda, isolated from garden soil, Mar. 2017, F. Versantvoort, JW 167004; ibid. JW 168007; Rijen, isolated from garden soil, Mar. 2017, G. & L. Schijvenaars, JW 94009. North Holland province, Hilversum, isolated from garden soil, Mar. 2017, S. Nieuwenhuijsen, JW 23021. Utrecht province, Amersfoort, isolated from garden soil, Mar. 2017, M. Kerssen, JW 125028; Amersfoort, isolated from garden soil, Mar. 2017, E., K. & O. de Jong Verpaalen, JW 241011; Amersfoort, isolated from garden soil, Mar. 2017, F. Wiegerinck, specimen CBS H-24102, culture CBS 145911 = JW 4017; Amersfoort, isolated from garden soil, Mar. 2017, T. & K. Wesselink, JW 191004; Bilthoven, isolated from garden soil, Mar. 2017, Y. El Ghazi, JW 220011; Utrecht, isolated from garden soil, Mar. 2017, J. Kooijmans, JW 63001.

**Figure 5.**
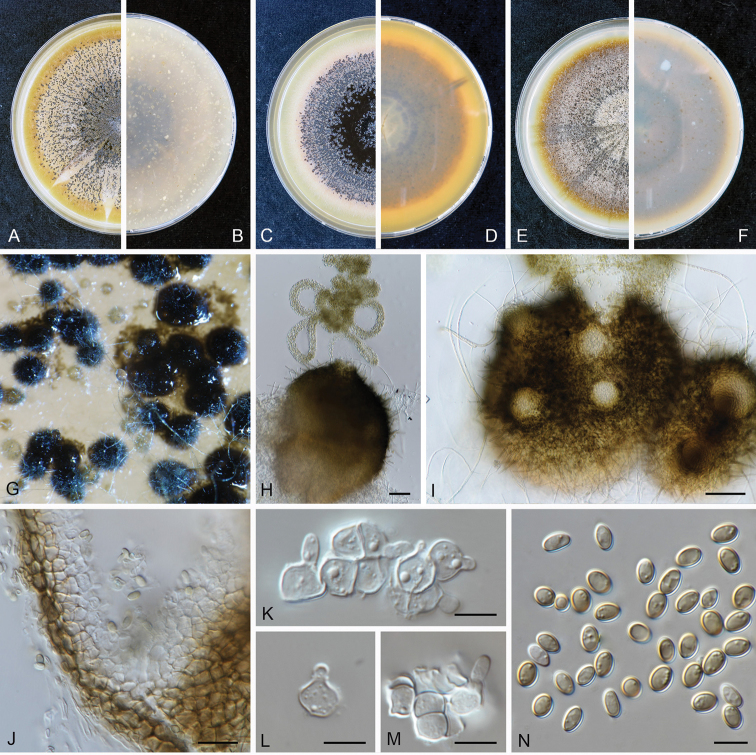
*Juxtiphoma kolkmaniorum* (CBS 146005). **A, B** Colony on OA (front and reverse) **C, D** colony on MEA (front and reverse) **E, F** colony on PDA (front and reverse) **G, H** pycnidium forming on OA**I** chlamydospores **J–L** conidiogenous cells **M** conidia. Scale bars: 100 μm (**G, H**); 10 μm (**I–M**).

##### Notes.

*Juxtiphoma kolkmaniorum* is very similar and phylogenetically close to *J. eupyrena*. However, based on the multi-gene phylogenetic analyses, *J. kolkmaniorum* forms a separate clade (Figure 1). Morphologically, *J. kolkmaniorum* has conidia slightly larger and with more guttules than those of *J. eupyrena* (3.5–7.5 × 2–3 μm, 1–3-guttulate vs. 4.2–5.6 × 1.8–2.4 μm, 2-guttulate, de Gruyter and Noordeloos 1992) and smaller chlamydospores (5.5–12 × 4–8 μm vs. 8–20 × 6–15 μm, de Gruyter and Noordeloos 1992).

#### Nothophoma brennandiae

Taxon classificationFungiPleosporalesDidymellaceae

Hern.-Restr., L. W. Hou, L. Cai & Crous sp. nov.

ED31092F-7A61-5A8A-BD8E-CF112EDB1A15

833198

Figure 6

##### Etymology.

*brennandiae* refers to Kristel Brennand who collected the soil sample from which the ex-type strain was isolated.

##### Typus.

The Netherlands. Limburg province, Ell, isolated from garden soil, Mar. 2017, K. Brennand (***holotype*** designated here CBS H-24103, living ex-type culture CBS 145912 = JW 53011).

*Conidiomata* pycnidial, superficial to semi-immersed, solitary to confluent, globose to subglobose, irregularly-shaped with age, brown, setose, especially near the ostioles, 155–350 × 100–300 μm; with 1–4 papillate ostioles; pycnidial wall pseudoparenchymatous, 3–6 layers, 13.5–21.5 μm thick, outer layers composed of brown, flattened polygonal cells. *Conidiogenous cells* phialidic, hyaline, smooth, ampulliform or somewhat isodiametric, 3–5 × 5–8 μm. *Conidia* ellipsoidal, broadly ellipsoidal to oblong, straight, thick- and smooth-walled, hyaline becoming brown, aseptate, 3–8.5 × 1.5–3 μm, 1–6-guttulate, minute. *Conidial matrix* sepia to brown vinaceous.

##### Culture characteristics.

Colonies after 7 d at 25 °C, on OA reaching 50–55 mm diam, aerial mycelium scarce, spore mass with grease-like appearance, dark brick to sepia, cinnamon to the edge, abundant production of confluent pycnidia, margin entire; reverse concentric rings umber to cinnamon. On MEA reaching 47–50 mm diam, aerial mycelium scarce, spore mass with grease-like appearance, dark brick to sepia, cinnamon to the edge, abundant production of confluent pycnidia, margin entire; reverse concentric rings umber to cinnamon. On PDA reaching 50–55 mm diam, aerial mycelium moderate to scarce, cottony, buff, spore mass with grease-like appearance, dark brick, ochreous to the edge, margin entire; reverse concentric rings dark brick to cinnamon. NaOH spot test negative on OA.

##### Additional specimen examined.

The Netherlands. North Holland province, Amsterdam, isolated from garden soil, Mar. 2017, J. van Dijk, JW 1066.

##### Notes.

In the phylogenetic tree *N. brennandiae* was close to *N. quercina* and *N. pruni* (Figure 1). Morphologically, *N. brennandiae* can be distinguished from *N. quercina* by having setose conidiomata with up to 4 ostioles, while in *N. quercina* conidiomata are glabrous with a single ostiole (Sydow and Sydow 1915; Aveskamp et al. 2010). Furthermore, conidia in *N. quercina* are larger and have less guttules (5.5–9 × 2.5–5 μm, 0–2(–3) guttules) (Sydow and Sydow 1915; Aveskamp et al. 2010). On the other hand, *N. pruni* is characterised by hyaline conidia (Chethana et al. 2019), while *N. brennandiae* produces conidia that turn brown with age.

**Figure 6.**
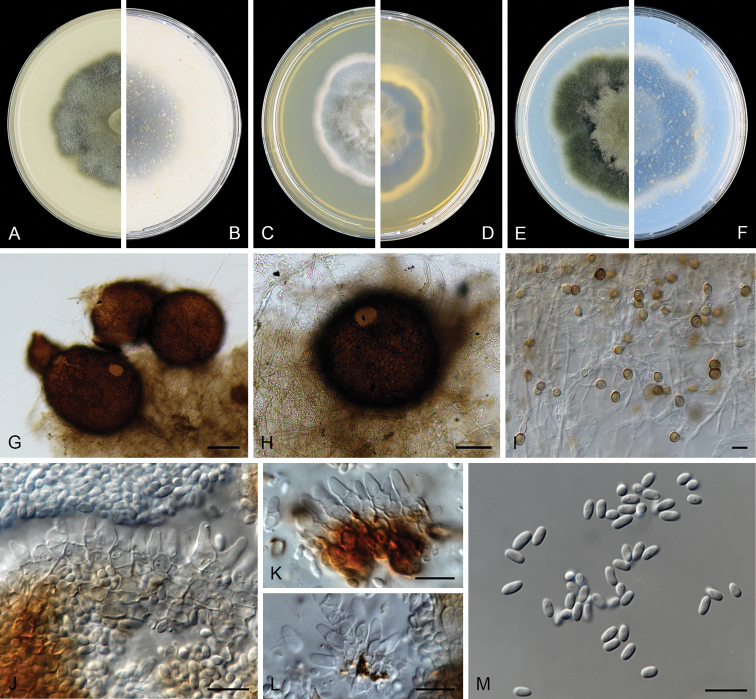
*Nothophoma brennandiae* (CBS 145912). **A, B** Colony on OA (front and reverse) **C, D** colony on MEA (front and reverse) **E, F** colony on PDA (front and reverse) **G** pycnidia forming on OA. **H, I** pycnidia **J** section of pycnidial wall **K–M** conidiogenous cells **N** conidia. Scale bars: 50 μm (**H, I**); 10 μm (**J**); 5 μm (**K–N**).

#### Paraboeremia rekkeri

Taxon classificationFungiPleosporalesDidymellaceae

Hern.-Restr., L. W. Hou, L. Cai & Crous sp. nov.

2D1ADC7E-658B-55A1-8696-E151FF5BDEC6

833199

Figure 7

##### Etymology.

*rekkeri* refers to Daan Rekker who collected the soil sample from which the ex-type strain was isolated.

##### Typus.

The Netherlands. Gelderland province, Geldermalsen, isolated from garden soil, Mar. 2017, D. Rekker (***holotype*** designated here CBS H-24107, living ex-type culture CBS 144955 = JW 172002).

*Conidiomata* pycnidial, superficial, scattered or aggregated, solitary or confluent, globose or subglobose, irregularly-shaped with age, buff to brown, covered with abundant mycelial outgrowths especially when young, 150–390 × 120–320 μm; 1–2 papillate or non-papillate ostioles; pycnidial wall pseudoparenchymatous, 3–7 layers, 17.5–37 μm thick, outer layers composed of brown, flattened polygonal cells, 10–21 μm diam. *Conidiogenous cells* phialidic, hyaline, smooth, globose, subglobose or ampulliform, 5–10 × 4.5–7.5 μm. *Conidia* ellipsoidal to oblong, thin- and smooth-walled, hyaline, aseptate, 3.5–5 × 2.5–3 μm, with 2(–3) large guttules. *Conidial matrix* pink.

##### Culture characteristics.

Colonies after 7 d at 25 °C, on OA reaching 75–80 mm diam, aerial mycelium floccose, saffron, vinaceous buff, pale olivaceous, margin regular; reverse concentric circles saffron, grey, olivaceous grey. On MEA reaching 55–60 mm diam, aerial mycelium floccose, margin irregular, pale olivaceous grey to whitish, orange near edge; reverse brown to dark brown, orange towards the periphery. On PDA reaching 70–75 mm diam, margin irregular, covered by felty aerial mycelium, buff, olivaceous grey towards periphery; reverse mouse, olivaceous towards periphery. NaOH spot test negative on OA.

##### Additional specimens examined.

Gelderland province, Culemborg, isolated from garden soil, Mar. 2017, H. van de Warenburg, JW 3018; Kapel-Avezaath, isolated from garden soil, Mar. 2017, A. Panneman, JW 79024; Meteren, isolated from garden soil, S. van Stuijvenberg, JW 132004; North Brabant province, Breda, isolated from garden soil, Mar. 2017, F. Wiegerinck, CBS 144949 = JW 4024; Breda, isolated from garden soil, Mar. 2017, F. Versantvoort, JW 167006; Zwanenburg, isolated from garden soil, Mar. 2017, J. Rebergen, JW 91008; North Holland province, Alkmaar, Mar. 2017, B. Verschoor, JW 13016, ibid. JW 13017 and JW13030; Utrecht province, Bilthoven, isolated from garden soil, Mar. 2017, H. Vos & S. Vos, JW 51014; Eemnes, isolated from garden soil, Mar. 2017, H.W. Vos, CBS 144950 = JW 6005; Hooglanderveen, isolated from garden soil, Mar. 2017, F. Rijpma, JW 25013; Utrecht, isolated from garden soil, R. van Zijl, JW 226002.

##### Notes.

*Paraboeremia rekkeri* formed a well-supported (1.0/100/96) distinct lineage in *Paraboeremia* (Figure 1). It is most closely related with *P. truiniorum*, another novel species collected from Dutch soil and described in the present study. However, *P. rekkeri* is distinguished by producing larger pycnidia (150–390 × 120–320 μm), with a thinner pycnidial wall (3–7 layers and 17.5–37 μm thick). Pycnidia in *P. truiniorum* are 160–420 × 135–430 μm, and have a wall of 7–11 layers and 40–70 μm thick.

**Figure 7.**
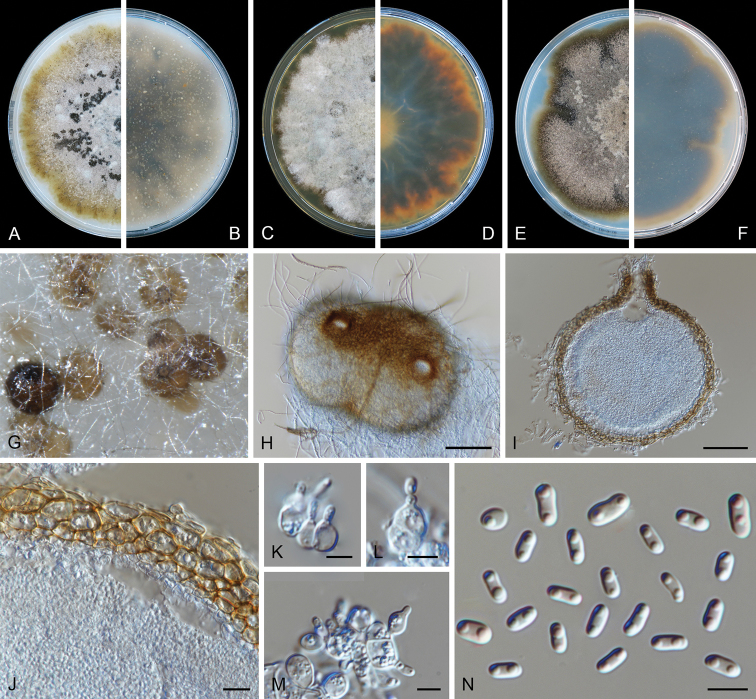
*Paraboeremia rekkeri* (CBS 144955). **A, B** Colony on OA (front and reverse) **C, D** colony on MEA (front and reverse) **E, F** colony on PDA (front and reverse) **G** pycnidia forming on OA**H** pycnidium **I** section of pycnidium **J** section of pycnidial wall **K–N** conidiogenous cells **O** conidia. Scale bars: 100 μm (**H**); 20 μm (**I**); 10 μm (**J**); 5 μm (**K–O**).

#### Paraboeremia truiniorum

Taxon classificationFungiPleosporalesDidymellaceae

Hern.-Restr., L. W. Hou, L. Cai & Crous sp. nov.

F8B7455D-5AFD-5C69-B571-5DDF3CE976C5

833201

Figure 8

##### Etymology.

*truiniorum* refers to Cuno & Tygo Truin who collected the soil sample from which the ex-type strain was isolated.

##### Typus.

The Netherlands. Gelderland province, Barneveld, Voorthuizen, isolated from garden soil, Mar. 2017, C. Truin & T. Truin (***holotype*** designated here CBS H-24108, living ex-type culture CBS 144952 = JW 47002).

*Conidiomata* pycnidial, superficial, scattered or aggregated, most solitary, globose or subglobose, confluent and irregularly-shaped with age, pale brown, thick-walled, covered with abundant mycelial outgrowths, 160–420 × 135–430 μm; 1-papillate or non-papillate ostioles, sometimes elongated to a short neck; pycnidial wall pseudoparenchymatous, 7–11 layers, 40–70 μm thick, outer layers composed of brown, flattened polygonal cells of 22–45.5 μm diam. *Conidiogenous cells* phialidic, hyaline, smooth, globose, subglobose, ampulliform or doliiform, 4.5–8.5 × 4–7 μm. *Conidia* ellipsoidal to oblong, thin- and smooth-walled, hyaline, aseptate, 3.5–5 × 2–3 μm, with (1–)2 large guttules. *Conidial matrix* whitish.

##### Culture characteristics.

Colonies after 7 d at 25 °C, on OA reaching 70–75 mm diam, aerial mycelium floccose, vinaceous buff to hazel, margin regular; reverse buff to olivaceous. On MEA reaching 65–70 mm diam, aerial mycelium felty, whitish, pale mouse grey toward periphery, margin regular; reverse dark brick to dark brown, with pale brown edge. On PDA reaching 75–80 mm diam, aerial mycelium felty, olivaceous buff to pale mouse grey, olivaceous toward periphery, margin irregular; reverse mouse grey, olivaceous toward periphery. NaOH spot test negative on OA.

##### Additional specimens examined.

The Netherlands, Gelderland province, Culemborg, isolated from garden soil, Mar. 2017, R. Fuld, JW 182014; The Netherlands. South Holland province, Alphen aan den Rijn, isolated from garden soil, Mar. 2017, K. Boutwell, CBS 144961 = JW 203021; The Netherlands. South Holland province, Gorinchem, isolated from garden soil, Mar. 2017, L. van Rosmalen, JW 270002; The Netherlands. Utrecht province, Utrecht, isolated from garden soil, Mar. 2017, L. van Rijnberk, JW 147025; The Netherlands. Utrecht province, Woerden, isolated from garden soil, Mar. 2017, L. Borsboom, JW 192003.

##### Notes.

Based on the phylogenetic analyses, *P. truiniorum* is represented by six isolates, forming a distinct lineage (Figure 1). *Paraboeremia truiniorum* is characterised by the dense mycelial outgrowths on its pycnidia. Both *P. truiniorum* and *P. rekkeri* are phylogenetically close to the well-known soil-borne species, *P. putaminum*. However, *P. putaminum* is distinguished from these two new species by producing smaller conidia (3.2–4.2 × 2–2.6 μm) with greenish guttules (Boerema et al 2004).

**Figure 8.**
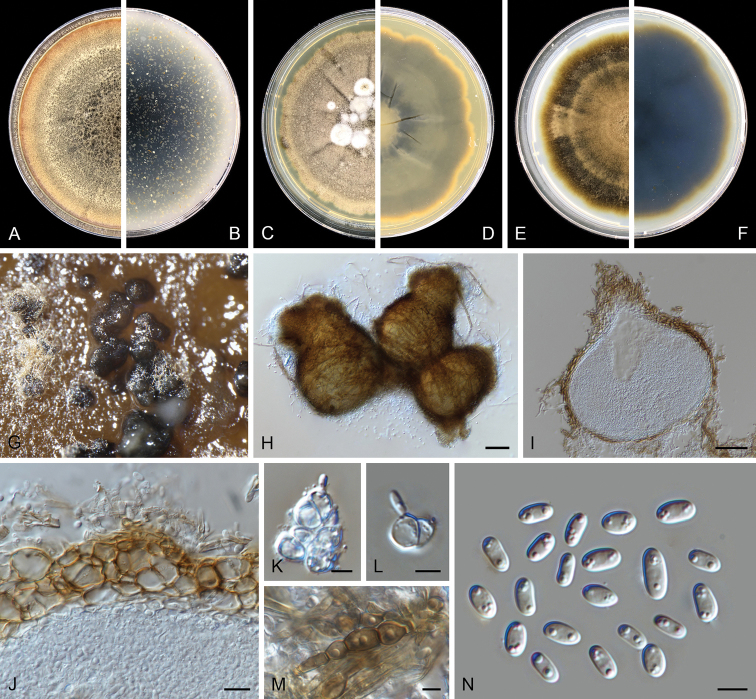
*Paraboeremia truiniorum* (CBS 144952). **A, B** Colony on OA (front and reverse) **C, D** colony on MEA (front and reverse) **E, F** colony on PDA (front and reverse) **G** pycnidia forming on OA**H** pycnidium **I** section of pycnidium **J** section of pycnidial wall **K–N** conidiogenous cells **O** conidia. Scale bars: 20 μm (**H**); 50 μm (**I**); 5 μm (**J–O**).

#### Stagonosporopsis stuijvenbergii

Taxon classificationFungiPleosporalesDidymellaceae

Hern.-Restr., L. W. Hou, L. Cai & Crous sp. nov.

0C4F7DF1-0BB7-5D4A-8D6B-1CABB52F5CE1

833203

Figure 9

##### Etymology.

*stuijvenbergii* refers to Simon van Stuijvenberg, who collected the soil sample from which the ex-type strain was isolated.

##### Typus.

The Netherlands. Gelderland province, Meteren, from garden soil, Mar. 2017, S. van Stuijvenberg (***holotype*** designated here CBS H-24109; living ex-type culture CBS 144953 = JW 132011).

*Conidiomata* pycnidial, produced on the agar surface, scattered or aggregated, solitary globose to subglobose, or 4–7(–10) confluent and irregularly-shaped, brownish, glabrous, ostiolate, 200–1000 × 195–930 μm; with 1–2 slightly papillate ostioles, sometimes elongated to a short neck; pycnidial wall pseudoparenchymatous, 4–5 layers, 6.5–35 μm thick, outer layers composed of brown, flattened polygonal cells, 9.5–33 μm diam. *Conidiogenous cells* phialidic, hyaline, smooth, globose, ampulliform or lageniform, 4.5–9 × 4–8 μm. *Conidia* ellipsoidal to oblong, smooth- and thin-walled, hyaline, aseptate, 3.5–6.5 × 2–3 μm, 1–2-guttulate. *Conidial matrix* whitish.

##### Culture characteristics.

Colonies after 7 d at 25 °C, on OA reaching 75–80 mm diam, floccose aerial mycelium, olivaceous to pale olivaceous, whitish to pink near the edge, margin regular; reverse iron grey. On MEA reaching 65–70 mm diam, margin regular, aerial mycelium floccose, vinaceous buff with olivaceous edge; reverse darker brown with olivaceous black edge, buff near the centre. On PDA reaching 70–75 mm diam, margin regular, covered by floccose aerial mycelium, olivaceous, olivaceous black towards periphery, with pinkish to pale brown edge; reverse iron-grey, buff towards periphery. NaOH spot test negative on OA.

##### Additional specimens examined.

The Netherlands, Gelderland province, Arnhem, from garden soil, Mar. 2017, D. Peters, JW 14003; Utrecht province, Utrecht, from garden soil, Mar. 2017, N. Francisca, JW 44014; Utrecht, from garden soil, Mar. 2017, P. de Koff, JW 33021.

##### Notes.

Phylogenetically, *S. stuijvenbergii* is most closely related to *S. weymaniae*, another novel species collected from Dutch soil in this study (Figure 1). However, *S. stuijvenbergii* is distinguishable from *S. weymaniae* by the colour and the size of its pycnidia, being brown and measuring 200–1000 × 195–930 μm in *S. stuijvenbergii*, whereas *S. weymaniae* produces whitish pycnidia, measuring 330–650 × 250–550 μm. Furthermore, *S. weymaniae* produces microconidia and chlamydospores, which were not observed in *S. stuijvenbergii.* Although there are several reports that *Stagonosporopsis* spp. could survive in soil for a short time (Vaghefi et al. 2016), this is the first record of a *Stagonosporopsis* species only known from soil (Domsch et al. 2007). *Stagonosporopsis stuijvenbergii* is represented by four strains isolated from different samples collected in Utrecht and Gelderland provinces.

**Figure 9.**
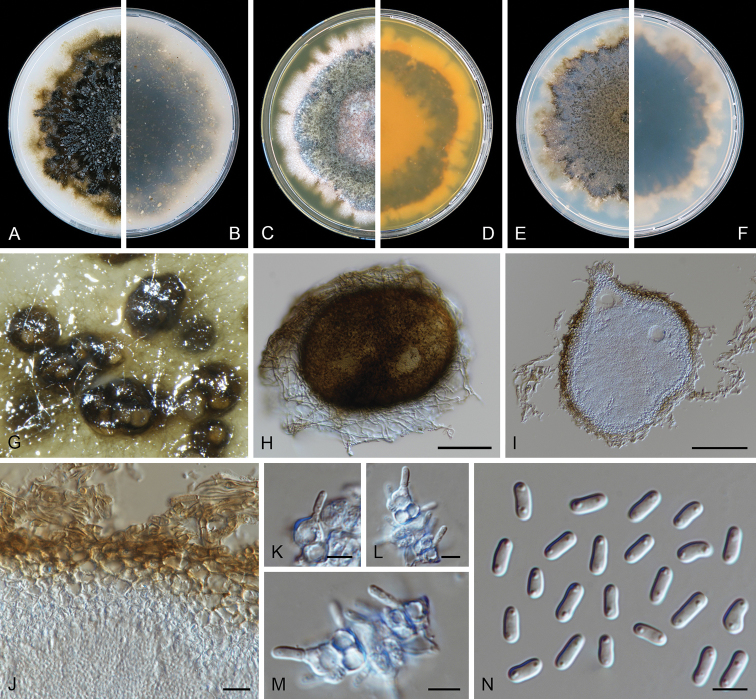
*Stagonosporopsis stuijvenbergii* (CBS 144953). **A, B** Colony on OA (front and reverse) **C, D** colony on MEA (front and reverse) **E, F** colony on PDA (front and reverse) **G** pycnidia forming on OA**H** pycnidia **I** ostiole **J–L** conidiogenous cells **M** stromatic hyphal aggregations **N** conidia. Scale bars: 50 μm (**H**); 10 μm (**I, M**); 5 μm (**J–L, N**).

#### Stagonosporopsis weymaniae

Taxon classificationFungiPleosporalesDidymellaceae

Hern.-Restr., L. W. Hou, L. Cai & Crous sp. nov.

FEC84CB9-4916-5A72-A05A-05189B939B0E

833204

Figure 10

##### Etymology.

*weymaniae* refers to Anna Weyman, who collected the soil sample from which the ex-type strain was isolated.

##### Typus.

The Netherlands. Utrecht province, Baarn, isolated from garden soil, Mar. 2017, A. Weyman (***holotype*** designated here CBS H-24110; living ex-type culture CBS 144959 = JW 201003).

*Conidiomata* pycnidial, semi-immersed or immersed, mostly solitary, scattered or aggregated, (sub-)globose, whitish to buff, glabrous, 330–650 × 250–550 μm; non-ostiolate or with a single, inconspicuous ostiole; pycnidial wall pseudoparenchymatous, 2–9 layers, 20–60 μm thick, outer layers composed of hyaline, flattened polygonal cells. *Conidiogenous cells* phialidic, hyaline, smooth, (sub-)globose to ampulliform, 4.5–7.5 × 4–7.5 μm. *Macroconidia* oblong, smooth- and thin-walled, hyaline, aseptate, 4–6.5(–8) × 2–3 μm, 1–3(–4)-guttulate, with one large central guttule or two large polar guttules. *Microconidia* produced in the same pycnidia with macroconidia, globose to subglobose, smooth, hyaline, aseptate, 3–4 × 2.5–3.5 μm, with a single, small guttule. *Conidial matrix* whitish. *Chlamydospores* unicellular, intercalary in chains, barrel-shaped, thick-walled, pale brown to green brown, guttulate, 9.5–14 × 11–16 μm diam.

##### Culture characteristics.

Colonies after 7 d at 25 °C, on OA reaching 70–75 mm diam, sparse aerial mycelium, buff to pale olivaceous with sparse olivaceous zones, darker grey near the centre, abundant production of buff pycnidia, margin regular; reverse pale olivaceous, olivaceous black near the centre. On MEA reaching 80–85 mm diam, margin regular, aerial mycelium floccose, yellow to vinaceous buff; reverse orange to olivaceous. On PDA reaching 75–80 mm diam, margin regular, covered by floccose aerial mycelium, centre vinaceous buff, dark olivaceous towards the periphery with production of buff pycnidia; reverse olivaceous black, olivaceous towards the periphery. NaOH spot test: pale reddish discolouration on OA plate.

##### Notes.

*Stagonosporopsis weymaniae* is phylogenetically closely related to *S. stuijvenbergii* (Figure 1). Morphological differences between *S. weymaniae* and *S. stuijvenbergii* are discussed under the latter species. *Stagonosporopsis weymaniae* together with *S. stuijvenbergii* formed a sister group with *S. bomiensis* and *S. papillata*, two plant pathogens from China (Chen et al. 2017). However, *S. weymaniae* differs from them by producing larger pycnidia [330–650 × 250–550 μm vs. 100–200 × 100–180 μm in *S. bomiensis* and (130–)200–280 × (100–)150–250 μm in *S. papillata*] and microconidia which are absent in *S. papillata* and *S. bomiensis* (Chen et al. 2017).

**Figure 10.**
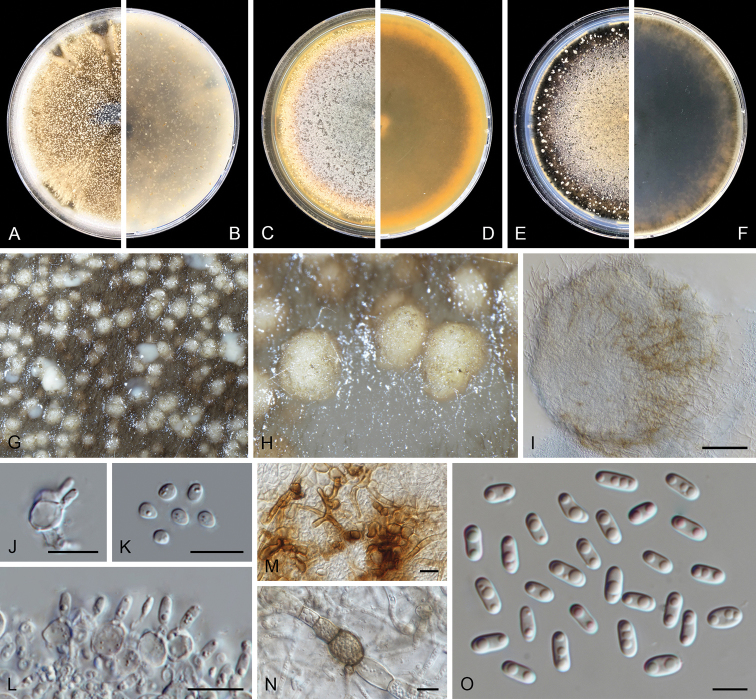
*Stagonosporopsis weymaniae* (CBS 144959). **A, B** Colony on OA (front and reverse) **C, D** colony on MEA (front and reverse) **E, F** colony on PDA (front and reverse) **G–I** pycnidia forming on OA**J, L** conidiogenous cells **K** subglobose conidia **M** stromatic hyphal aggregations **N** chlamydospores **O** oblong conidia. Scale bars: 100 μm (**I**); 10 μm (**J–N**); 5 μm (**O**).

#### Vandijckomycella

Taxon classificationFungiPleosporalesDidymellaceae

Hern.-Restr., L. W. Hou, L. Cai & Crous gen. nov.

53B36B04-4D9B-5F2A-8F7C-E046793133BC

833205

##### Etymology.

Named in honour of José F.T.M. van Dijck, who was elected as the first female President (2015–2018) of the Royal Dutch Academy of Arts and Sciences (KNAW).

##### Type species.

*Vandijckomycella joseae* Hern.-Restr., L.W. Hou, L. Cai & Crous.

*Conidiomata* pycnidial, superficial on the surface of the agar, solitary or confluent, globose to lageniform, covered by hyphal outgrowths, ostiolate, pycnidial wall pseudoparenchymatous, with 3–9 layers. *Conidiogenous cells* phialidic, hyaline, smooth, globose or ampulliform. *Conidia* hyaline, smooth- and thin-walled, aseptate, ovoid, oblong or ellipsoidal, with 2–4 polar guttules.

#### Vandijckomycella joseae

Taxon classificationFungiPleosporalesDidymellaceae

Hern.-Restr., L. W. Hou, L. Cai & Crous sp. nov.

F0D67FCC-EBBF-581B-9BAD-8232E67A1D62

833208

Figure 11

##### Etymology.

Named in honour of the first female President (2015–2018) of the Royal Dutch Academy of Arts and Sciences (KNAW), José F.T.M. van Dijck, who collected the soil sample from which the ex-type strain was isolated.

##### Typus.

The Netherlands. North Holland province, Amsterdam, isolated from garden soil, Mar. 2017, J.F.T.M. van Dijk (***holotype*** designated here CBS H-24112; living ex-type culture CBS 143011 = JW 1073).

*Conidiomata* pycnidial, produced on the agar surface, scattered or aggregated, solitary, (sub-)globose, confluent and irregularly-shaped with age, pale brown, covered in abundant long and thin mycelium hair, 150–340 × 130–250 μm; with 1–2 slightly papillate or non-papillate ostioles, sometimes elongated to a short neck; pycnidial wall pseudoparenchymatous, 3–5 layers, 13–25 μm thick, outer layers composed of brown, flattened, polygonal cells of 10–23 μm diam. *Conidiogenous cells* phialidic, hyaline, smooth, globose, ampulliform, lageniform or subglobose, 5–8(–9.5) × 4–8 μm. *Conidia* ellipsoidal to oblong, smooth- and thin-walled, hyaline, aseptate, 3.5–5.5 × 2–2.5 μm, (1–)2(–3)-guttulate. *Conidial matrix* whitish.

##### Culture characteristics.

Colonies after 7 d at 25 °C, on OA reaching 75–80 mm diam after 7 d, covered by woolly aerial mycelium, concentric circles, pale olivaceous grey, pink, pale greenish grey, whitish near the edge, margin regular; reverse concentric circles dark brown, pale brown, orange, and pale olivaceous. On MEA reaching 75–80 mm diam, aerial mycelium woolly, margin regular, pale olivaceous grey; reverse dark brown, reddish towards the periphery. On PDA reaching 75–80 mm diam, margin regular, covered by felty aerial mycelium, pale olivaceous grey or olivaceous grey, with whitish parts near the centre or through the plate; reverse zonate, orange to reddish, brown and yellow. NaOH spot test: a coral discolouration on OA.

##### Additional specimen examined.

The Netherlands. North Holland province, Amsterdam, isolated from garden soil, Mar. 2017, J.F.T.M. van Dijk, CBS 144948 = JW 1068.

##### Notes.

The new genus *Vandijckomycella* is introduced to accommodate two new species isolated from soil samples which form an independent lineage in Didymellaceae, being clearly separated from other genera (Figure 1). Based on the phylogenetic analysis, *V. joseae* forms a distinct lineage which is distant from the nearest species *V. snoekiae*, and chiefly differs on *tub2* and *rpb2* sequences. Morphological differences between *V. joseae* and *V. snoekiae* are discussed under the latter species. *Vandijckomycella joseae* is characterised by producing pycnidia with longer whitish hyphal outgrowths, and with elongated necks.

**Figure 11.**
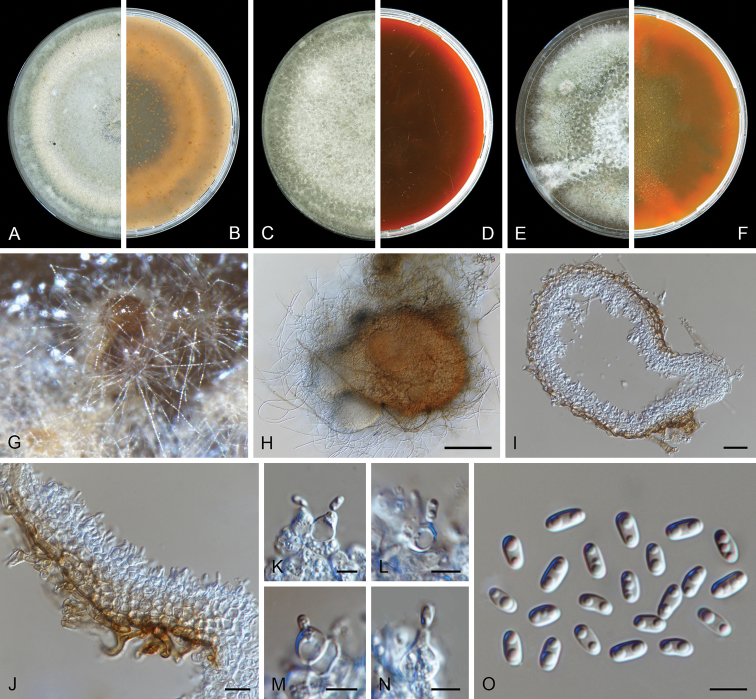
*Vandijckomycella joseae* (CBS 143011). **A, B** Colony on OA (front and reverse) **C, D** colony on MEA (front and reverse) **E, F** colony on PDA (front and reverse) **G, H** pycnidia forming on OA**I, J** section of pycnidial wall **K–N** conidiogenous cells **O** conidia. Scale bars: 100 μm (**H**); 20 μm (**I**); 10 μm (**J**); 5 μm (**K–O**).

#### Vandijckomycella snoekiae

Taxon classificationFungiPleosporalesDidymellaceae

Hern.-Restr., L. W. Hou, L. Cai & Crous sp. nov.

EC229B27-79DD-51C1-BD80-7B37EEA8A92C

833207

Figure 12

##### Etymology.

*snoekiae* refers to Rana Marit Ida Snoek who collected the soil sample from which the ex-type strain was isolated.

##### Typus.

The Netherlands. Utrecht province, Utrecht, isolated from garden soil, Mar. 2017, R.M.I. Snoek (***holotype*** designated here CBS H-24111, living ex-type culture CBS 144954 = JW 149017).

*Conidiomata* pycnidial, superficial on the agar or covered under a thick mycelial layer, scattered or aggregated, mostly solitary, globose to subglobose, sometimes confluent, ellipsoidal, dark brown, covered by abundant long hyphal outgrowths, 150–650(–850) × 145–600(–730) μm; ostioles inconspicuous; pycnidial wall pseudoparenchymatous, 5–9 layers, 37–58.5 μm thick, outer layers composed of brown, flattened polygonal cells, 10–23 μm diam. *Conidiogenous cells* phialidic, hyaline, smooth, globose, ampulliform or lageniform, 5–8.5 × 5–7.5 μm. *Conidia* oblong, smooth- and thin-walled, hyaline, aseptate, 4–6.5 × 2–2.5 μm, with two small polar guttules. *Conidial matrix* whitish.

##### Culture characteristics.

Colonies after 7 d at 25 °C, on OA reaching 50–55 mm diam after 7 d, covered by floccose aerial mycelium, pink to grey, darker grey near the centre, margin regular; reverse black near the centre, yellow towards the periphery. On MEA reaching 50–55 mm diam, aerial mycelium floccose to cottony, buff with some mouse grey zones, margin regular; reverse orange with some radial yellow lines and some black zones. On PDA, reaching 45–50 mm diam, covered by floccose aerial mycelium, vinaceous grey to pale olivaceous, olivaceous grey near the centre, margin irregular; reverse buff to orange, black near the centre. NaOH spot test on OA: pale reddish discolouration.

##### Notes.

Morphologically, *V. snoekiae* differs from its closest phylogenetic neighbour *V. joseae* in the size of its pycnidia and the number of ostioles. *Vandijckomycella snoekiae* produces larger pycnidia with inconspicuous ostioles, measuring 150–650(–850) × 145–600(–730) μm, while *V. joseae* produces pycnidia with 1–2 ostioles, measuring 150–340 × 130–250 μm. In addition, *V. snoekiae* produces conidia with less and smaller guttules than *V. joseae* (2 guttules, vs. 1–3 large guttules).

**Figure 12.**
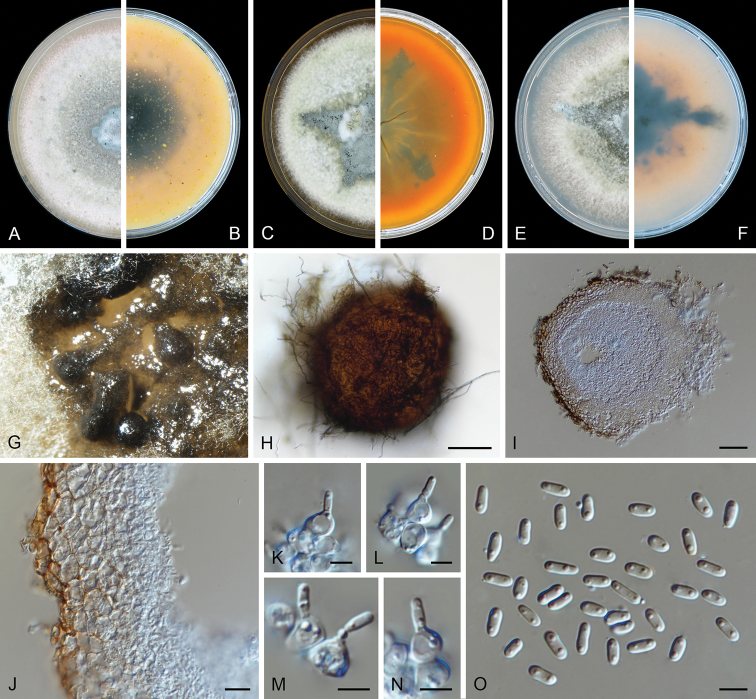
*Vandijckomycella snoekiae* (CBS 144954). **A, B** Colony on OA (front and reverse) **C, D** colony on MEA (front and reverse) **E, F** colony on PDA (front and reverse) **G, H** pycnidia forming on OA**I, J** section of pycnidial wall **K–N** conidiogenous cells **O** conidia. Scale bars: 100 μm (**H**); 50 μm (**I**); 10 μm (**J**); 5 μm (**K–O**).

#### Xenodidymella weymaniae

Taxon classificationFungiPleosporalesDidymellaceae

Hern.-Restr., L. W. Hou, L. Cai & Crous sp. nov.

9A0B3880-71A9-571A-9B1A-A7E1D9F5BF6E

833209

Figure 13

##### Etymology.

*weymaniae* refers to Anna Weyman who collected the soil sample from which the ex-type strain was isolated.

##### Typus.

The Netherlands. Utrecht province, Baarn, isolated from garden soil, Mar. 2017, A. Weyman (***holotype*** designated here CBS H-24113; living ex-type culture CBS 144960 = JW 201005).

*Conidiomata* pycnidial, semi-immersed on the agar, mostly confluent, sometimes solitary, scattered or aggregated, subglobose or ellipsoidal, irregularly-shaped when confluent, dark brown, ostiolate, glabrous or with long hyphal outgrowths around the ostiole, 100–700 × 100–400(–590) μm; with 1–2(–6) ostioles, papillate or elongated into a long neck, up to 113 μm in length; pycnidial wall pseudoparenchymatous, 3–5 layers, 17–45 μm thick, outer layers composed of pale brown to brown, flattened polygonal cells of 10–35 μm diam. *Conidiogenous cells* phialidic, hyaline, smooth, sub-globose, ampulliform or lageniform, 4.5–8 × 4–6.5 μm. *Conidia* oblong, smooth- and thin-walled, hyaline, aseptate, 4–6(–8) × 2–2.5 μm, with two small, polar guttules. *Conidial matrix* whitish.

##### Culture characteristics.

Colonies after 7 d at 25 °C, on OA reaching 55–60 mm diam, aerial mycelium floccose near the centre, flat towards the periphery, pale olivaceous to whitish, black pycnidia visible near the centre, margin regular; reverse buff to salmon, pale olivaceous towards the periphery. On MEA reaching 40–45 mm diam, aerial mycelium felty, sectors with cottony mycelium, white, buff to pale olivaceous, margin regular; reverse yellow to orange, dark brown and pale grey near the centre. On PDA reaching 45–60 mm, aerial mycelium floccose, whitish in the centre, honey towards the periphery, margin regular; reverse concentric circles dark brown in centre, orange, yellow, buff towards the periphery. NaOH spot test negative on OA.

##### Notes.

*Xenodidymella weymaniae* formed a distinct branch basal to *X. applanata* (Figure 1). Morphologically, *X. weymaniae* could be clearly differentiated from *X. applanata* in pycnidial and conidial characteristics. In *X. weymaniae* pycnidia are dark brown, ostioles have elongated necks, 100–700 × 100–400(–590) μm, and conidia are oblong, with 2 small polar guttules. In *X. applanata* pycnidia are pale brown, with single, slightly papillate ostioles, 85–175 × 60–145 μm, and ellipsoidal to ovoid conidia, with several guttules (Chen et al. 2015). Furthermore, the two species can also be distinguished from the NaOH spot test on OA medium (negative vs. pale reddish discoloration). This is the first record of a *Xenodidymella* species isolated from soil (Boerema et al. 2004; Chen et al. 2015, 2017).

**Figure 13.**
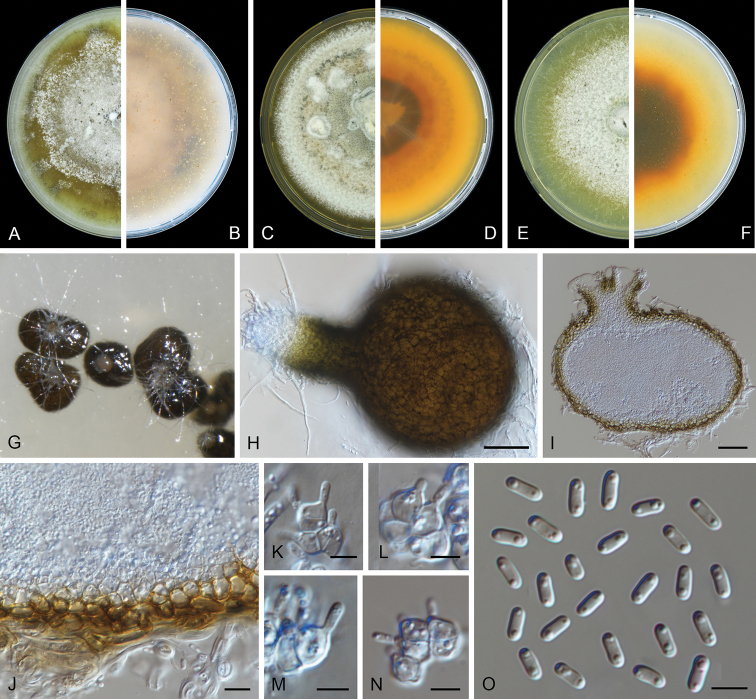
*Xenodidymella weymaniae* (CBS 144960). **A, B** Colony on OA (front and reverse) **C, D** colony on MEA (front and reverse) **E, F** colony on PDA (front and reverse) **G, H** pycnidia forming on OA**I, J** section of pycnidial wall **K–N** conidiogenous cells **O** conidia. Scale bars: 50 μm (**H**); 20 μm (**I**); 10 μm (**J**); 5 μm (**K–O**).

## Discussion

During the present Citizen Science project which focused on Dutch soil fungi, numerous unknown species of filamentous and yeast fungi were described (Crous et al. 2017, 2018; Groenewald et al. 2018; Giraldo et al. 2019). As part of the project, we focused on investigating species diversity of Didymellaceae from soil samples obtained in the Netherlands.

As one of the largest families in the fungal kingdom, at least 26 genera are accepted in Didymellaceae (Chen et al. 2015, 2017; Valenzuela-Lopez et al. 2018) and more than 5400 species names are recorded in MycoBank to date (Crous et al. 2004), representing 4.2 % of the 120000 accepted fungal species. However, only around 30 ubiquitous species have been found in the soil environment as saprophytes, mainly in *Ascochyta*, *Boeremia*, *Didymella*, *Epicoccum* and *Phoma* (Boerema et al. 2004; Aveskamp et al. 2008, 2010; Chen et al. 2015, 2017). In our set of samples, we found 20 species distributed in 10 genera i.e. *Ascochyta*, *Calophoma*, *Didymella*, *Juxtiphoma*, *Nothophoma*, *Paraboeremia*, *Phomatodes*, *Stagonosporopsis*, *Vandijckomycella* and *Xenodidymella*. However, we did not find any species of *Phoma* and *Epicoccum*, probably due to the media used for primary isolation, and also because of the taxonomical changes that have been suffered by many species of both genera in recent years (Aveskamp et al. 2010, Chen et al. 2015)

*Paraboeremia* and *Juxtiphoma* were the most dominant genera. Species of *Paraboeremia* are more common on plants than in soil, except for *P. putaminum*, which is regarded as a widespread soil-borne fungus isolated from the subterranean parts of various herbaceous and woody plants (de Gruyter and Noordeloos 1992; Boerema et al. 2004). In the present study this species was the most abundant species, being recovered from 29 soil samples from 19 cities. Besides *P. putaminum*, one isolate was identified as *P. litseae*, which was previously only known on diseased leaves of *Litsea* from China (Jiang et al. 2016). In addition, two new species were described, namely *P. rekkeri* found in Gelderland, North Brabant, North Holland and Utrecht provinces and *P. truiniorum* found in South Holland and Utrecht provinces.

The second most abundant species was *Juxtiphoma eupyrena*. The monotypic genus *Juxtiphoma* was recently introduced to accommodate *Phoma eupyrena* (Valenzuela-Lopez et al. 2018), a cosmopolitan soil-inhabiting fungus, which may cause damping-off of seedlings of herbaceous and woody plants (de Gruyter and Noordeloos 1992; Boerema et al. 2004; Morgan-Jones and Burch 1988), but was also reported as an opportunistic human pathogen (Bakerspigel et al. 1981). Furthermore, a new species was introduced in this genus as *Juxtiphoma kolkmaniorum* which includes 12 of our soil isolates (JW) and one strain (CBS 527.66) isolated from soil in a wheat field in Germany.

Among our isolates we found *Phomatodes nebulosa*, *Didymella macrostoma* and *D. pomorum* which are plurivorous and cosmopolitan species often isolated from soil (Boerema 1993; de Gruyter et al. 1993; Farr and Rossman 2019). Interestingly, we found two species identified as plant pathogens that had not been previously reported from soil, including *Ascochyta syringae* and *Calophoma clematidis-rectae*. *Ascochyta syringae* causes ascochyta blight of Lilac (*Syringa vulgaris*) in America, Australia and Europe (Farr and Rossman 2019), while *Calophoma clematidis-rectae* is known on *Clematidis* spp. in the Netherlands (Aveskamp et al. 2010). In addition, *Stagonosporopsis* is recognised mainly as a phytopathogenic genus on different plant hosts (Marin-Felix et al. 2019). However, we found two new species from soil, namely *S. stuijvenbergii* and *S. weymaniae*. Other new species described include *A. benningiorum*, *D. degraaffiae*, *D. kooimaniorum*, *N. brennandiae*, *V. joseae*, *V. snoekiae*, and *X. weymaniae*.

These findings suggest that species of Didymellaceae are also widely distributed in soil. Previous studies have revealed that many pathogens survive in soil by producing resting bodies (Dorenbosch 1970; Aveskamp et al. 2008), such as *A. pinodes* (currently: *Didymella pinodes*) and Phoma medicaginis var. pinodella (currently: *Didymella pinodella*) that produce chlamydospores or brown, thick-walled, swollen hyphae associated with sporocarps, which allow these species to survive in the soil for several years after the decay of their host tissues (Tivoli and Banniza 2007). On the other hand, some harmless saprobes in this family have also been observed to switch from an opportunistic to pathogenic lifestyle once in contact with the appropriate host (Aveskamp et al. 2008). Therefore, it is probable that the described new taxa are dormant in soil, remaining able to infect hosts under favourable conditions, especially species from phytopathogenic genera such as *S. stuijvenbergii*, *S. weymaniae*, *N. brennandiae* and *X. weymaniae*. However, considering that soil is a dynamic and multifunctional system and that the fungal community and its distribution are closely related to various living organisms such as plants, animals and insects, it was difficult to establish whether the species found in this study were true soil inhabitants or transferred to the soil via external vectors (such as worms, nematodes, etc.). Whether these new taxa originate from other habitats, or could change to pathogenic or endophytic lifestyles given the right conditions, remains to be determined. Furthermore, as the soil ecosystem is very complex and each type of soil and location may possess its own unique species diversity, the true diversity of Didymellaceae and their role in soil remains to be elucidated.

Recently, additional research based on cultivation-independent and cultivation-dependent methods has revealed that Didymellaceae species present in various soil environments are more diverse than one might have expected (Bell et al. 2014; Nallanchakravarthula et al. 2014; Li et al. 2016; Miao et al. 2016; Zhang et al. 2016a, 2016b; Chen et al. 2017; Nagano et al. 2017). Although recent high-throughput methods have detected a higher diversity of soil fungi compared with those based on culture-dependent methods, it is not possible to identify these taxa to species or even to genus level, as ITS sequence data alone are insufficient for species delimitation in most fungal families including Didymellaceae. Therefore, cultivation-dependent methods are still indispensable in the investigation of true species diversity of Didymellaceae based on additional loci such as *rpb2* and *tub2* obtained from cultivated isolates.

In summary, results of our study revealed the presence of a large number of unknown species and even a novel genus in soil, illustrating that this substrate is an important source for the discovery of novel taxa, and demonstrating that species diversity of Didymellaceae in soil is considerably greater than current estimates.

## Supplementary Material

XML Treatment for Ascochyta benningiorum

XML Treatment for Didymella degraaffiae

XML Treatment for Didymella kooimaniorum

XML Treatment for Juxtiphoma kolkmaniorum

XML Treatment for Nothophoma brennandiae

XML Treatment for Paraboeremia rekkeri

XML Treatment for Paraboeremia truiniorum

XML Treatment for Stagonosporopsis stuijvenbergii

XML Treatment for Stagonosporopsis weymaniae

XML Treatment for Vandijckomycella

XML Treatment for Vandijckomycella joseae

XML Treatment for Vandijckomycella snoekiae

XML Treatment for Xenodidymella weymaniae
